# Molecular differences of angiogenic versus vessel co-opting colorectal cancer liver metastases at single-cell resolution

**DOI:** 10.1186/s12943-023-01713-1

**Published:** 2023-01-24

**Authors:** Johannes Robert Fleischer, Alexandra Maria Schmitt, Gwendolyn Haas, Xingbo Xu, Elisabeth Maria Zeisberg, Hanibal Bohnenberger, Stefan Küffer, Laure-Anne Teuwen, Philipp Johannes Karras, Tim Beißbarth, Annalen Bleckmann, Mélanie Planque, Sarah-Maria Fendt, Peter Vermeulen, Michael Ghadimi, Joanna Kalucka, Tiago De Oliveira, Lena-Christin Conradi

**Affiliations:** 1 Department of General, Visceral and Pediatric Surgery, University Medical Center Göttingen, Robert-Koch-Straβe 40, 37075 Göttingen, Germany; 2 Department of Cardiology and Pneumology, University Medical Center Göttingen, Robert-Koch-Straβe 40, 37075 Göttingen, Germany; 3 German Center for Cardiovascular Research (DZHK), Partner Site, Göttingen, Germany; 4 Institute of Pathology, University Medical Center Göttingen, Robert-Koch-Straβe40, 37075 Göttingen, Germany; 5 Department of Oncology, Antwerp University Hospital (UZA), Drie Eikenstraat 655, 2650 Edegem, Belgium; 6 Department of General- and Visceral Surgery, Raphaelsklinik Münster, Loerstraße 23, 48143 Münster, Germany; 7 Department of Medical Bioinformatics, University Medical Center Göttingen, Goldschmidtstraße 1, 37077 Göttingen, Germany; 8 Department of Medicine A, Hematology, Oncology, and Pneumology, University Hospital Münster, 48149 Münster, Germany; 9 Laboratory of Cellular Metabolism and Metabolic Regulation, VIB-KU Leuven Center for Cancer Biology, VIB, Leuven, Belgium; 10 Laboratory of Cellular Metabolism and Metabolic Regulation, Department of Oncology, KU Leuven and Leuven Cancer Institute (LKI), Leuven, Belgium; 11 Translational Cancer Research Unit, GZA Hospitals, Sint-Augustinus, University of Antwerp, Antwerp, Belgium; 12 Department of Biomedicine, Aarhus University, Høegh-Guldbergsgade 10, 8000 Aarhus C, Denmark; 13 Steno Diabetes Center Aarhus, Aarhus University Hospital, Aarhus, Denmark

**Keywords:** Colorectal cancer liver metastases, Histopathological growth patterns, Vessel co-option, Sprouting angiogenesis, Glycolysis, WNT signalling, Pentose phosphate pathway

## Abstract

**Background:**

Colorectal cancer liver metastases (CRCLM) are associated with a poor prognosis, reflected by a five-year survival rate of 14%. Anti-angiogenic therapy through anti-VEGF antibody administration is one of the limited therapies available. However, only a subgroup of metastases uses sprouting angiogenesis to secure their nutrients and oxygen supply, while others rely on vessel co-option (VCO). The distinct mode of vascularization is reflected by specific histopathological growth patterns (HGPs), which have proven prognostic and predictive significance. Nevertheless, their molecular mechanisms are poorly understood.

**Methods:**

We evaluated CRCLM from 225 patients regarding their HGP and clinical data. Moreover, we performed spatial (21,804 spots) and single-cell (22,419 cells) RNA sequencing analyses to explore molecular differences in detail, further validated in vitro through immunohistochemical analysis and patient-derived organoid cultures.

**Results:**

We detected specific metabolic alterations and a signature of WNT signalling activation in metastatic cancer cells related to the VCO phenotype. Importantly, in the corresponding healthy liver of CRCLM displaying sprouting angiogenesis, we identified a predominantly expressed capillary subtype of endothelial cells, which could be further explored as a possible predictor for HGP relying on sprouting angiogenesis.

**Conclusion:**

These findings may prove to be novel therapeutic targets to the treatment of CRCLM, in special the ones relying on VCO.

**Supplementary Information:**

The online version contains supplementary material available at 10.1186/s12943-023-01713-1.

## Introduction

Colorectal cancer (CRC) five-year survival rates range from up to 90% in cases of locally limited tumors to 14% when disease has spread to distant organs [1, 2]. By far, the liver is the most common site of metastasis, observed in up to 75% of metastatic cases [3]. To date, different CRCLM HGPs have been defined and were shown to be of prognostic relevance for a patient’s outcome [4–6]. The three predominant CRCLM HGPs observed are the desmoplastic HGP (dHGP), the replacement HGP (rHGP) and the rarely observed pushing HGP (pHGP) [6]. CRCLM having a dHGP have better survival rates compared to other HGPs [4, 5]. Besides their morphologic characteristics, HGPs also distinctly differ in their main mechanism of blood supply, having relevant clinical implications regarding anti-angiogenic treatment [7, 8]. Both dHGP and pHGP CRCLM rely primarily on sprouting angiogenesis (SA) [9, 10], a process commonly driven by angiogenic factors such as hypoxia-inducible factor-1 (HIF-1) and vascular endothelial growth factor (VEGF) [11, 12]. In contrast, CRCLM displaying the rHGP predominantly utilize vessel co-option (VCO) to secure their oxygen and nutrient supply [8, 9] and increased cancer cell motility and adhesion have been suggested as driving factors of this process [7, 13]. Compared to angiogenic HGPs, rHGP CRCLM show reduced susceptibility to anti-angiogenic treatment such as the VEGF-inhibitor bevacizumab [7].

To improve therapeutic strategies targeting VCO in the rHGP, in-depth molecular characterization of the interplay between cancer cells and the tumor microenvironment is essential. Therefore, the identification of detectable biomarkers prior to resection for differing HGPs is critical given that they respond differently to anti-angiogenic treatment [7]. In this single-center study, we analysed a cohort of 225 patients according to their CRCLM HGP and correlated these data to their clinical outcomes. Since renin–angiotensin–aldosterone system (RAAS) inhibition was suggested to soften the fibrotic CRCLM stroma and to have an impact on tumor mechanical stiffness [14], we evaluated the overall survival (OS) of a subgroup of CRCLM patients under anti-RAAS medication. We also performed spatial (spaRNA-seq) and single-cell RNA sequencing (scRNA-seq) analyses to determine cellular and molecular signatures involved in the different CRCLM HGPs. We detected specific metabolic alterations and a signature of WNT signalling activation in metastatic cancer cells related to the rHGP phenotype. Of note is that in the corresponding healthy liver of dHGP CRCLM, we identified a predominantly expressed capillary subtype, which could be further explored as a possible predictor for dHGP. Our findings suggest to further exploit glycolysis and the WNT signalling pathway as possible targets for the treatment of rHGP CRCLM.

## Methods

### Patient cohort and histopathological characteristics

#### Patient samples

For analysis of clinical aspects, we established a retrospective cohort of 225 patients (summarized in Supplementary Table 1) who underwent CRCLM resection between 1995 and 2015 at the University Medical Center Göttingen (UMG), Germany. From eight patients, two CRCLM were available, resulting in a total of 233 CRCLM. The study was approved by the UMG Ethics Committee (25/3/17). For PDOs, surgically fresh resected CRCLM tissue was derived from 10 patients between 2020 and 2021, of which five exhibited rHGP and five exhibited dHGP (Supplementary Table 12), as assessed retrospectively. Samples were collected at the UMG under ethical approval of the UMG (25/3/17 and 23/4/22).

#### HGP scoring of CRCLM

HGP scoring of CRCLM was performed according to the international consensus guidelines [10] by two independent evaluators using H&E slides containing an area of tumor-liver interface. The HGP were estimated as percentage proportions of the visible tumor-liver interface. In the case of mixed HGP, all expressed HGP were rounded to 5% values. For further analysis, the CRCLM were categorized according to the main mechanism of blood supply, which the respective HGP relies on. Therefore, dHGP and pHGP were summed up as a SA group, whereas the rHGP was classified as a VCO group. For clinical analysis, the exact cut-off values for each group are displayed in the figures. For IHC analysis, a cut-off value of ≥ 80% was chosen to include CRCLM in the SA or VCO group. For the MS-based metabolic profiling, cut-off values of 50% were used.

### Spatial transcriptomics

#### Slide preparation, staining and imaging

The Visium Spatial Gene Expression for the FFPE Kit (10 × Genomics, PN-1000338) was used to generate sequencing libraries. Prior to section placement, tissue adhesion was assured using the Visium Tissue Section Test Slides (10 × Genomics, PN-1000347) and the RNA-extraction of FFPE blocks was performed using RNeasy FFPE Kit (Qiagen, #73,504). For RNA quality evaluation, including determination of DV200, the Agilent 2100 Bioanalyzer system with Agilent RNA 6000 Nano Kit (5067–1511) was used. All samples reached DV200 of at least 50%. Sections of 5 µm thickness were cut from the FFPE CRCLM from six patients (3 dHGP and 3 rHGP) and placed on the capture areas of the Visium Spatial Gene Expression Slide (10 × Genomics, PN-2000233). Each capture area with a size of 6.5 × 6.5 mm contains roughly 5000 unique gene expression spots with a diameter of 55 µm. Following the manufacturer´s protocol, the sections were deparaffinized, H&E-stained, coverslipped and imaged at 40 × magnification with the Glissando Objective Imaging scanner (Objective Imaging Ltd, Cambridge, UK). Tissue permeabilization and construction of sequencing libraries were performed following the manufacturer’s protocol (10 × Genomics, PN-1000338).

#### Reverse transcription, spatial library preparation and sequencing

Libraries were sequenced using the DNBSEQ™ technology (BGI). Therefore, DNA Nanoballs (DNB) were generated and all samples were loaded on one flow cell using the DNBSEQ-G400 High-throughput Sequencing Set (BGI, 1,000,016,970). Two samples were pooled together on one sequencing lane. The MGISEQ-2000 sequencer (BGI) was used with the following settings: Paired-end run with 28 cycles for read1 (encoding spatial Barcode and UMI), 50 cycles for read2 (encoding the ligated probe insert), 10 cycles for the i5 index and 10 cycles for the i7 index (identifying each sample) (PE28 + 50 + 10 + 10). The sequencing depth was 300 M reads per lane which equals a sequencing depth of 150 M reads per sample.

#### Spatial transcriptomics data processing

After sequencing, libraries were de-multiplexed, mapped to the human transcriptome and aligned to overlaying H&E images using SpaceRanger software (10 × Genomics) and the manual alignment tool (LoupeBrowser v5.1.0, 10 × Genomics). All further steps were performed using the UniApp (Unicle Biomedical Data Science, Belgium).

#### Quality control

Across all patients, 22,272 tissue-covered spots were detected. Detailed quality metrics for every sample are shown in Supplementary Table 3. For quality filtering, spots with an expression of less than 200 genes/spot, ambiguous expression of canonical marker genes or location on folded tissue were excluded, resulting in 21,804 high quality spots included for further analysis.

#### Graph-based clustering of single samples and cluster annotation

For clustering the spots of every single sample, data was auto-scaled and dimensional reduction was performed using principal component analysis (PCA). The first 30 PCAs were visualized in t-Distributed Stochastic Neighbour Embedding (t-SNE) with a t-SNE perplexity of 60 and a learning rate of 200. Graph-based clustering was performed to cluster the spots according to their gene expression profile (clustering-resolution = 0.8, k-nearest neighbours = 10). Since every spot captures the transcriptomics of several spatially overlaying cells, predominating cell types in every cluster were determined using canonical marker genes. In the case that clusters could not be identified by expression of canonical marker genes, clusters were annotated according to their morphological appearance on the H&E slide. Clusters were further investigated by identifying the top 50 uniquely upregulated marker genes for each determined cluster.

#### Upset plots and Jaccard similarity PCA

Upset plots were generated using the R package “UpsetR” (v1.4.0). Jaccard similarity analysis [15] was calculated using a custom R script (provided by Unicle Biomedical Data Science, Belgium).

#### Data visualization

The UniApp (Unicle Biomedical Data Science, Belgium) was used for data visualization, including t-SNE plots, spatial plotting of spots on the H&E slide, heatmaps and dot plots. Heatmaps are based on cluster-averaged gene expression to account for cell-to-cell transcriptomic stochastics. In all heatmaps, data were auto-scaled for visualization.

#### Pathway mapping

First, differential expression analysis between two clusters (cancer areas dHGP vs. cancer areas rHGP) was performed using limma (pmid25605792), as described previously (pmid29608177). Genes with adjusted p-value < 0.05 from selected canonical pathways derived from the KEGG database (WNT signalling pathway, M19428; Glycolysis/Gluconeogenesis, M11521; PPP, M1386) were shown. Logfold (A) of selected genes was scaled using the following equation:

$$-0.5+\frac{1}{1+{e}^{20*-(A)}}$$

For mapping, a colour scale with expression -0.5 to 0.5 was used.

### Single-cell RNA sequencing analysis

#### scRNA-seq sample collection, library preparation and sequencing

CRCLM and normal, non-transformed hepatic tissue (located as distant to the metastases as possible) from six patients with CRCLM (three dHGP and three rHGP) (Supplementary Table 13) were used for scRNA-seq. The HGP was determined retrospectively using H&E slides covering the tumor-liver interface at the margin of sample collection. For depletion of dead cells, specimens underwent single-cell dissociation with the gentleMACs Octo Dissociator (Miltenyi Biotec,130–095-937), cryopreservation at -80 °C and magnetic-activated cell sorting (MACS) with Basic MicroBeads (Miltenyi Biotec, 130–048-001). Using the Chromium single-cell sequencing solution (10 × Genomics), we performed single-cell separation, cDNA amplification and library construction. In detail, single-cell gel bead-in emulsions were generated using the 10 × Chromium Single Cell Controller followed by library construction with the Chromium Next GEM Single cell 3´ GEM Library & Gel Bead Kit v3.1 (10 × Genomics, PN-1000123). The HS DNA Bioanalyzer with the dsDNA Qubit Kit was used to measure cDNA and library concentrations.

Libraries were sequenced using the DNBSEQ™ technology (BGI), generating DNA Nanoballs (DNB) on a flow cell using the DNBSEQ-G400 High-throughput Sequencing Set (BGI, 1,000,016,970). Two samples were pooled together on one sequencing lane. The MGISEQ-2000 sequencer (BGI) was used with the following settings: DNBseqPE100 + 100 + 10. The sequencing depth was 300 M reads per lane which equals a sequencing depth of 150 M reads per sample.

#### Quality control and data normalization

Raw data was demultiplexed, aligned to the human genome and implemented in a gene count matrix using CellRanger software (10 × Genomics). Detailed quality metrics are shown in Supplementary Table 14. For quality filtering, genes detected in fewer than three cells and cells with less than 150 detected genes per cell were excluded to catch quiescent low transcriptome cell types such as EC. Here, we first excluded cells in which > 70% of transcripts were derived from mitochondria (to include tumor cells with low RNA quantity, likely experiencing leakage phenomena [16]), followed by manual quality control applied after the initial clustering. Therefore, clusters lacking expression of canonical marker genes or clusters expressing marker genes from distinct cell lineages (doublet removal) were removed in repetitive steps, totalizing 22,419 cells.

#### Graph-based clustering and cluster annotation

For clustering, data was auto-scaled and dimensional reduction was performed using PCA. The first 20 PCAs were visualized in t-Distributed Stochastic Neighbour Embedding (t-SNE). Graph-based clustering was performed to cluster the spots according to their gene expression profile (clustering-resolution = 1). The uniquely upregulated genes per cluster were identified using the function in the UniApp (Unicle Biomedical Data Science, Belgium) and carefully reviewed. Finally, the expression of canonical marker genes was also explored, so that major cell types for each cluster could be identified.

#### Bootstrap analysis

We applied hierarchical clustering with Euclidean distance and average linkage. We estimated the confidence of all branches of the tree by the bootstrap resampling approach from the R-package *pvclust *[17]. To ensure that biologically relevant branches that could not be directly resolved by bootstrapping were statistically separable (e.g. capillaries and and ACLs), we performed pair-wise differential analysis and confirmed that these clusters had at least ten genes that exceed a 0.2 log_2_fold enrichment with a FDR corrected *p* value < 0.05.

#### Data visualization

UniApp (Unicle Biomedical Data Science, Belgium) was used for data visualization, including t-SNE plots, heatmaps and dot plots. Heatmaps were based on cluster-averaged gene expression to account for cell-to-cell transcriptomic stochastics. In all heatmaps, data was auto-scaled for visualization. For cluster correlation, heatmaps were calculated with the value of tiles for self correlation defined as non applicable, to highlight the other correlations (in this cases no scaling was used). To ensure data accessibility to non-bioinformaticians, reproducibility and resource value, we made our scRNA-seq data available for further exploration via an interactive webtool: https://unicle.life/portals/. Using this tool, users can interactively visualize gene expression and clustering on t-SNE, search marker genes for all subclusters and export gene expression data.

#### Gene set enrichment analysis

GSEA was used as implemented in the clusterProfiler package by comparing gene expression signatures between groups (pmid22455463). A collection of gene sets (PID, KEGG, REACTOME, BP) selected from the Molecular Signature Database (MsigDB v7.5.1 downloaded from https://www.gsea-msigdb.org/gsea/msigdb/https://www.gsea-msigdb.org/gsea/msigdb/ was used. GSEA scores were calculated for sets having at least 10 detected genes; all other parameters were default.

### Integrative analysis of spaRNA-seq and scRNA-seq

#### Feature engineering

Gene sets containing the 30 and 150 most enriched marker genes of the identified ACL phenotype of ECs (scRNA-seq) were established and used for feature engineering (Supplementary Table 20). Therefore, relative expression of those gene sets in each spot of the spaRNA-seq data set was computed and spatially displayed on the H&E overlay using the function in the UniApp.

### In vitro* functional assays*

#### Primary CRCLM patient-derived organoid isolation and culture

Establishment and culturing of PDOs were performed according to Sato et al. 2011 [18]. Briefly, PDOs were cultured in Matrigel (Corning) using advanced DMEM/F12 medium supplemented with 10 mM Hepes, 1X Glutamax, 1X penicillin/streptomycin (10,000 U) (all from Gibco), 10 mM nicotinamide (Sigma), 10 µM SB202190 (MedChem Express, Monmouth Junction, USA), 1X B27 (Gibco), 1X N2 supplement (Gibco), 500 nM A83-01 (Tocris), 10% Noggin CM (made in house), 20% R-spondin conditioned media (CM) (made in house), 1.25 mM N-acetylcysteine (Sigma) and 50 ng/ml human EGF (Gibco). 100 µg/ml Primocin (InvivoGen) was added to the medium only during extracting and passaging PDOs for the first time. 10 µM Y-27632 (Adooq Biosciences, Irvine, USA) were added to the medium after extraction and seeding of PDOs. PDOs were respectively stratified for HGP based on H&E slides from the corresponding CRCLM.

#### TOP/FOP-Flash reporter assay

TOP/FOP-Flash reporter plasmids containing intact or mutated Tcf/Lef binding sites (Addgene, 12,456; Addgene, 12,457) and Renilla plasmids (Promega, E2241) were co-transfected in H1299 cells using Lipofectamine3000 (Invitrogen, USA; 15,282,465). Transfected H1299 cells were treated with PDO supernatant (R-Spondin-reduced PDO tumor media conditioned by PDOs for two and seven days) for 16 h. The Dual Luciferase® Reporter Assay System (Promega, E1910) was used to measure luciferase activities. The assay was performed in triplicates. TOP/FOP-Flash values were normalized to the Renilla values and to the number of viable cells in corresponding PDOs, generating the TOP/FOP-Flash ratio.

#### Histology and immunostaining

First, FFPE samples of CRCLM were cut in 2-µm slides, deparaffinized in xylol (Carl Roth, Karlsruhe; Germany) and further subjected to descending series of ethanol (Carl Roth) (from 100 to 70% ethanol) following standard H&E staining protocol (3 min Meyer’s hematoxylin (Merck, Darmstadt, Germany) 2 × 2 min wash in destilated water, 1 min eosin (Merck) incubation and final dehydration in increasing ethanol series (from 70 to 100% ethanol) and xylol (all Carl Roth)). For IHC, FFPE tissue was obtained from tissue micro-arrays blocks. Here, samples were deparaffinized in xylol, underwent a decreasing series of ethanol and were washed in deionized water. Slides were then heated for 20 min at 100 °C in a steamer (Braum, Melsungen, Germany) in Tris–EDTA (pH 8,5). After which, samples were incubated for 15 min in 3% H_2_O_2_ (in deionized water) to block endogenous peroxidase activity and blocked with 5% bovine serum albumin (BSA) diluted in PBS for 10 min before washing with Tris-buffered saline containing 0.1% TritonX100 (TBST). Samples were then incubated with the primary antibodies in an antibody diluent (Zytomed, ZUC025-100) with the following time periods and dilutions: LDHA (Thermo Fisher Scientific (TA500568), 1:200, 2 h); DKK1 (Bio-Rad (AHP1156) 1:100, 1 h), Ki67 (Invitrogen (MA5-14,520) 1:500, 1 h); Hif1α (Acris Antibodies (AP20633PU-N) 1:200, 1 h). After incubation, samples were washed two times for 5 min in TBST. Afterwards, samples were incubated with secondary antibodies. For AMPKα staining, samples were incubated for 30 min at room temperature (RT) in rabbit anti-goat Immunglobulins/Horse Raddish Peroxidase (HRP), diluted in TBST (1:200). For LDHA and DKK1 staining, samples were incubated in Labelled Polymer Anti-Rabbit/HRP (Dako North America, CA, USA (K4002)) for 30 min and then washed two times each for 5 min in TBST. Bright-DAB (ImmunoLogic, NL) was applied for 8 min on the samples before being immersed for 1 min in hematoxylin and further washed in deionized water. Finally, samples were dehydrated by incubation in an ascending ethanol series and xylol for 2 min. For mounting, Vitro-Clud® (Langenbrinck, Germany) was applied before adding a cover glass.

#### RNA isolation and qRT-PCR

PDOs were harvested for RNA extraction in PBS, centrifuged at RT and the pellet was processed using the *Quick*™-RNA Miniprep Kit (Zymo Research; R1054). RNA samples were reverse-transcribed to complementary DNA (cDNA), and subsequently, qPCR was performed as a one-step reaction using the SensiFAST™ SYBR® No-ROX One-Step Kit (Meridian Bioscience; BIO-72005) and the CFX384 real-time PCR system (Bio-rad). Results were obtained from three technical replicates per PDO line and normalized to the expression of Cyclophilin. Primers were produced by IDT™ (Integrated DNA Technologies BVBA, Belgium) and are listed in Supplementary Table 21.

#### Western blot

PDOs were harvested for protein extraction by mechanical destruction in PBS, centrifuged and the pellet incubated for 10 min in cell recovery solution (Corning, USA; 354,253). Pellets were lysed using modified RIPA buffer (50 mM Tris pH 7.8, 250 mM NaCl, 30 mM EDTA, 30 mM EGTA, 25 mM Sodium Pyrophosphate, 1% Triton X100, 0.5% NP-40, 10% Glycerol, 1 mM DTT) supplemented with protease inhibitor (Complete Mini Tables EDTA-free, Roche) and phosphatase inhibitor (PhosStop tablets, Roche) and then ruptured by passing through an insulin syringe (BD Microfine, BD, Germany). Lysates were centrifuged for 10 min at 4 °C and supernatants used for Western blot analysis. 30 µg protein per sample were subjected to 8% SDS-PAGE and transferred to 0.45 µm PVDF-membranes (Cytiva, 10,600,023). Membranes were blocked for 30 min at RT with 3% Notfat Dry Milk (Carl Roth, T145.3) prior to overnight incubation with the following primary antibodies: active β-Catenin 1:4000 (Millipore, #05–665), total β-Catenin 1:2000 (Cell Signaling Technology, #9587) and HSC70 1:2000 (Santa Cruz Biotechnology, #sc-7298). Membranes were then incubated with HRP-conjugated anti-rabbit IgG 1:10,000 (Thermo Fisher Scientific, G-21234) or anti-mouse IgG 1:2000 (Thermo Fisher Scientific, A-10668) secondary antibodies for 30 min at RT. Blots were developed with SuperSignal West Pico Chemoluminescence Substrate (Thermo Fischer Scientific, 15,513,766) and imaged with the ImageQuant LAS 4000 mini. The intensity of the bands was measured using ImageJ (NIH, Bethesda, MD) software. For quantification, intensity of the bands was normalized to a HSP70 loading control and p-values were calculated by comparing the HGPs.

### Bulk transcriptomics analysis

#### Gene set enrichment analysis – dnLef and ACL signature

The (GSE151165) [19] data set was the only currently available bulk sequencing data set stratifying CRCLM for HGPs by browsing NIH GEO DataSets for the terms “Colorectal cancer liver metastases” and “HGP”. Raw RNA-seq count tables from the (GSE151165) data set were normalized using the TMM method [20] from the edgeR package in bioconductor (version 3.14) [20, 21]. For analysis of the dnLef signature, all samples from normal adjacent liver were excluded from the analysis. For analysis of the ACL signature, all samples from CRCLM were excluded from the analysis. Read counts from CRCLM or adjacent normal liver (six rHGP and nine dHGP) were compared using the gene set enrichment analysis software (GSEA, University of California San Diego and Broad Institute, USA) [22, 23] (version 4.1.0). Genes were ranked according to differences in expression between the two classes using the signal-to-noise ratio as the ranking metric. The permutation type was set to “gene set” and the number of permutations was set to 1000. The normalized enrichment score (NES) calculated by the GSEA displays the level of over-representation of the defined gene set at the top or bottom of the ranked list, normalized in regard to the size of the gene set.

To create a customized gene signature, metabolism-associated genes downregulated upon induction of a dominant-negative isoform of Lef1 in DLD-1 colon cancer cells [24] (Supplementary Table 11) were compiled and expression was analysed in CRCLM from the GSE151165 [19] dataset (cancer tissue). Expression of the ACL signature (150 highest enriched genes), defined as described below (Supplementary Table 20), was analysed in adjacent healthy liver from the same data set.

### Metabolomics

#### Mass spectrometry and metabolic profiling

Metabolites extraction was performed, in a mixture ice/dry ice, by a cold two-phase methanol–water–chloroform extraction [25, 26]. The samples were resuspended in 800 μl of precooled methanol/water (5/3) (v/v) and 200 µL of 13C yeast internal standard. Afterwards, 500 μl of precooled chloroform was added to each sample. Samples were vortexed for 10 min at 4 °C and then centrifuged (max. speed, 10min, 4 °C). The methanol–water phase containing polar metabolites was separated and dried using a vacuum concentrator at 4 °C overnight and stored at −80 °C. For the detection of polar metabolites by LC–MS, a Dionex UltiMate 3000 LC System (Thermo Scientific) with a thermal autosampler set at 4 °C, coupled to a Q Exactive Orbitrap mass spectrometer (Thermo Scientific) was used for the separation of metabolites. Samples were resuspended in 70 µL of water and 10 µL of sample were injected, the separation of metabolites was achieved with a flow rate of 0.25 ml/min, at 40 °C, on a C18 column (Acquity UPLC HSS T3 1.8 μm 2.1 × 100 mm). A gradient was applied for 40 min (solvent A: 0 H2O, 10 mM tributyl-amine, 15 mM acetic acid—solvent B:Methanol) to separate the targeted metabolites (0 min: 0% B, 2 min: 0% B, 7 min:37% B, 14 min: 41% B, 26 min: 100% B, 30 min: 100% B, 31 min: 0% B; 40 min: 0% B). The MS operated in negative full scan mode (m/z range: 70–900) using a spray voltage of 4.9 kV, capillary temperature of 320 °C, sheath gas at 50.0, auxiliary gas at 10.0. Data was collected and analyzed using the Xcalibur software (Thermo Scientific). Results were finally normalized by protein content and 13C yeast internal standard. 

### Quantification and statistical analysis

In addition to bioinformatical approaches described above for spaRNAseq and scRNAseq, all other statistical analyses were performed using GraphPad Prism 8.2.1 software (GraphPad Holdings LLC, USA), unless stated otherwise. For survival analyses, the log-rank test (Mantel-Cox) was used to calculate p values between groups and the Kaplan–Meier method was used to plot survival curves. Multivariate Cox Proportional Hazards analysis was performed with the variables dHGP (100%), pUICC, age, gender and localization using software R (version 3.6.3, URL https://www.R-project.org, Vienna, Austria), with package *survival*. Results were considered statistically significant when *p* values < 0.05.

## Results

### Primary tumors localized in the rectum are more likely to develop rHGP CRCLM resulting in a worse prognosis

**Fig. 1:**
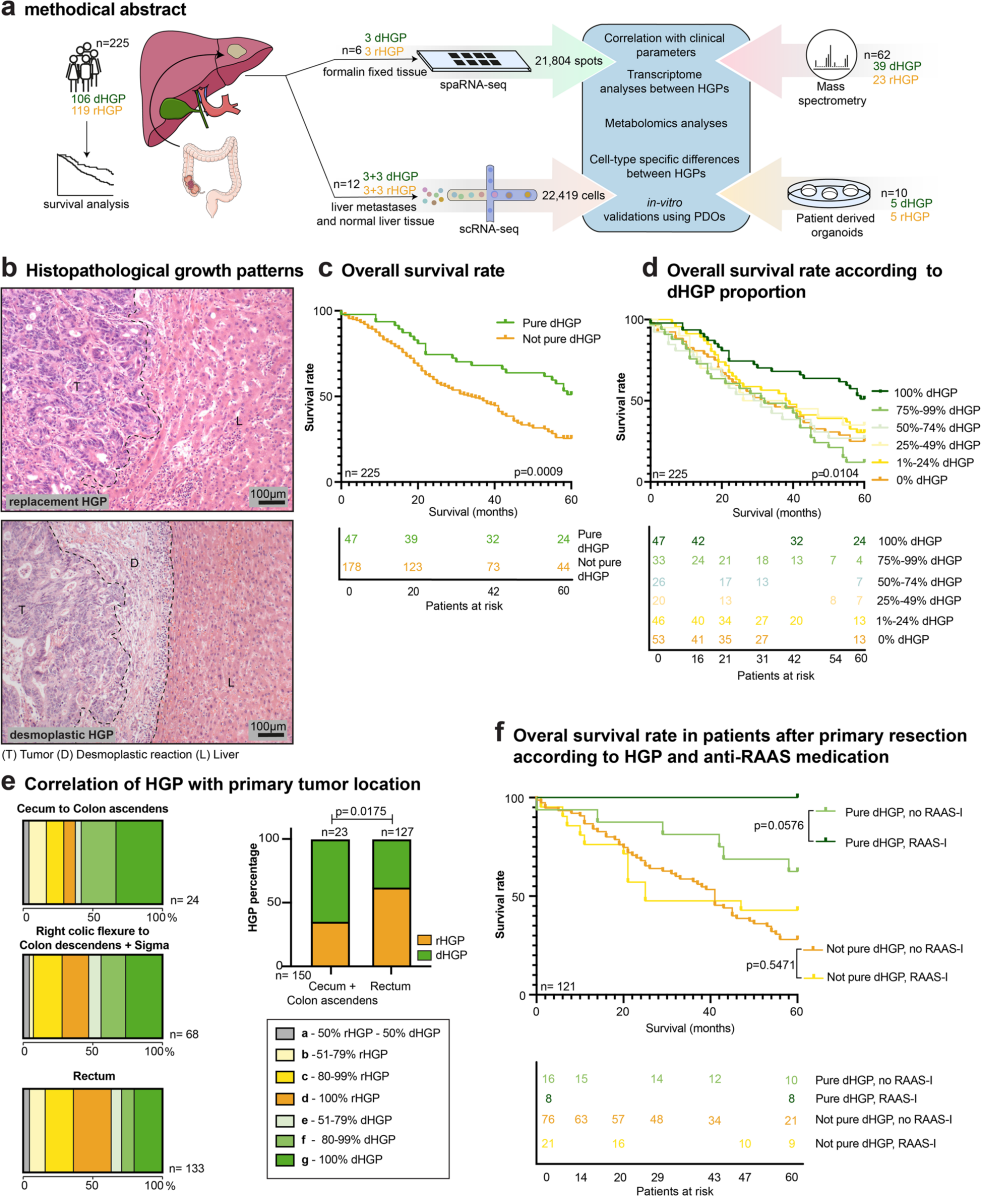
Primary tumors localized in the rectum are more likely to develop rHGP CRCLM. (**a**) Graphical abstract of the experimental workflow. (**b**) Representative H&E images of rHGP and dHGP. T: tumor; L: liver; D: desmoplastic rim. (**c**) Kaplan–Meier curves: OS after CRCLM resection depending on the HGP (*n* = 225). (**d**) Kaplan–Meier curves: sub-stratified OS after CRCLM resection depending on the percentage of dHGP (*n* = 225). (**e**) Left box charts: HGP distribution of CRCLM in the context of the localization of the primary tumor. Right bar plot: predominant HGP (after exclusion of CRCLM with 50% of each HGP), in the context of the localization of the primary tumor (*p* value by chi-square test). (**f**) Kaplan–Meier curves: OS after CRCLM resection depending on the HGP and the application of therapeutic RAAS inhibition (RAAS-I) in the pre-treatment-naive subgroup (*n* = 121). P values were calculated by two-sided log-rank test

We established a local cohort of CRCLM from 225 patients (Fig. 1a, Supplementary Table 1a) and classified CRCLM according to their HGPs (Fig. 1b). Importantly, the distribution of both HGPs was equally present in the cohort (Fig. S1a). CRCLM exclusively showing the dHGP were termed “*pure* dHGP”. Consequently, CRCLM that exhibited variable percentages of other HGPs (replacement or pushing) were named “*not pure* dHGP”. We next performed OS and multivariate cox proportional hazards analyses, which revealed a positive prognostic value of the *pure* dHGP in comparison to the *not pure* dHGP in our cohort (Fig. 1c and Supplementary Table 1b). Especially the subgroup of 121 patients who were pre-treatment-naive (no systemic anti-tumor treatment, including chemotherapy and/or antibodies such as anti-VEGF and anti-EGFR for 6 months prior to CRCLM resection) contributed to this trend (Fig. S1b, c). Further stratification according to different proportion percentages of dHGP clearly showed that 100% dHGP is associated with better survival than all subgroups with lower dHGP percentages (Fig. 1d). Subsequently, CRCLM HGPs were categorized according to their predominant type of blood supply. Thus, dHGP and pHGP were combined as CRCLM relying on SA, whereas rHGP was categorized as CRCLM relying on VCO.

We then evaluated whether different CRCLM HGPs would correlate with the localization of their respective primary tumors. Surprisingly, we found that primary tumors located in the rectum exhibited a higher rate of rHGP CRCLM. In contrast, CRCLM derived from primary tumors located in the cecum and colon ascendens showed a higher rate of dHGP and pHGP. A significant association was found when comparing these groups, excluding seven CRCLM with balanced mixed HGPs (50% dHGP and 50% rHGP) (Fig. 1e). No correlation was found when examining the relation between time of metastasis or gender with the HGP (Figure S1d, e).

In CRCLM, anti-RAAS medication is associated with an improved response to anti-angiogenic therapy by reducing activity of the metastases-associated fibroblasts, and thereby, reducing stiffness of the metastases [14]. Considering the distinct stromal composition in CRCLM with different HGPs [27], we evaluated the impact of anti-RAAS medication on the OS of patients with different HGPs CRCLM. For this purpose, we included 53 patients receiving a baseline treatment with two different anti-RAAS medications, namely angiotensine-converting enzyme inhibitors and angiostensine II receptor I antagonist, and performed survival analyses by comparing *pure* dHGP versus *not pure* dHGP. Interestingly, therapeutic RAAS inhibition showed a clear trend towards improved OS within the group of patients with *pure* dHGP, whilst the group of patients with a *not pure* dHGP exhibited no differences in OS rates (Fig. S1f). Similar findings were obtained when displaying the same analysis in the subgroup of 121 pre-treatment-naive patients. Strikingly, in this subgroup, all patients who were treated with anti-RAAS medication and displayed a *pure* dHGP survived the five-year observation period after CRCLM resection, whereas no other subgroup reached this outcome (Fig. 1f). Moreover, when similar analyses were performed in systemically pre-treated patients (chemotherapy and/or antibodies such as anti-VEGF and anti-epidermal growth factor receptor within 6 months prior to CRCLM resection), this effect on the survival rate was not observed (Fig. S1g).

Collectivey, these results show that CRCLM derived from tumors in the rectum are more likely to exhibit the rHGP, whilst tumors located in the cecum and colon ascendens presumably give rise to dHGP CRCLM. Moreover, anti-RAAS medication improves survival in *pure* dHGP patients, and especially those who are pre-treatment-naive.

### Spatial transcriptomic analysis of cancer areas reveals HGP-specific features

To further evaluate the histological architecture and molecular features of HGP CRCLM, their tumor microenviroment and surrounding hepatic tissue, we conducted spaRNA-seq. Formalin-fixed-paraffin-embedded (FFPE) tissue was derived from six different patients (Supplementary Table 2), of which three presented with the rHGP (PT36, PT44, PT54) and three showed the dHGP (PT55, PT61, PT68). After quality control, 21,804 tissue-covered spots across all patients were included in the analysis (Supplementary Table 3).

**Fig. 2:**
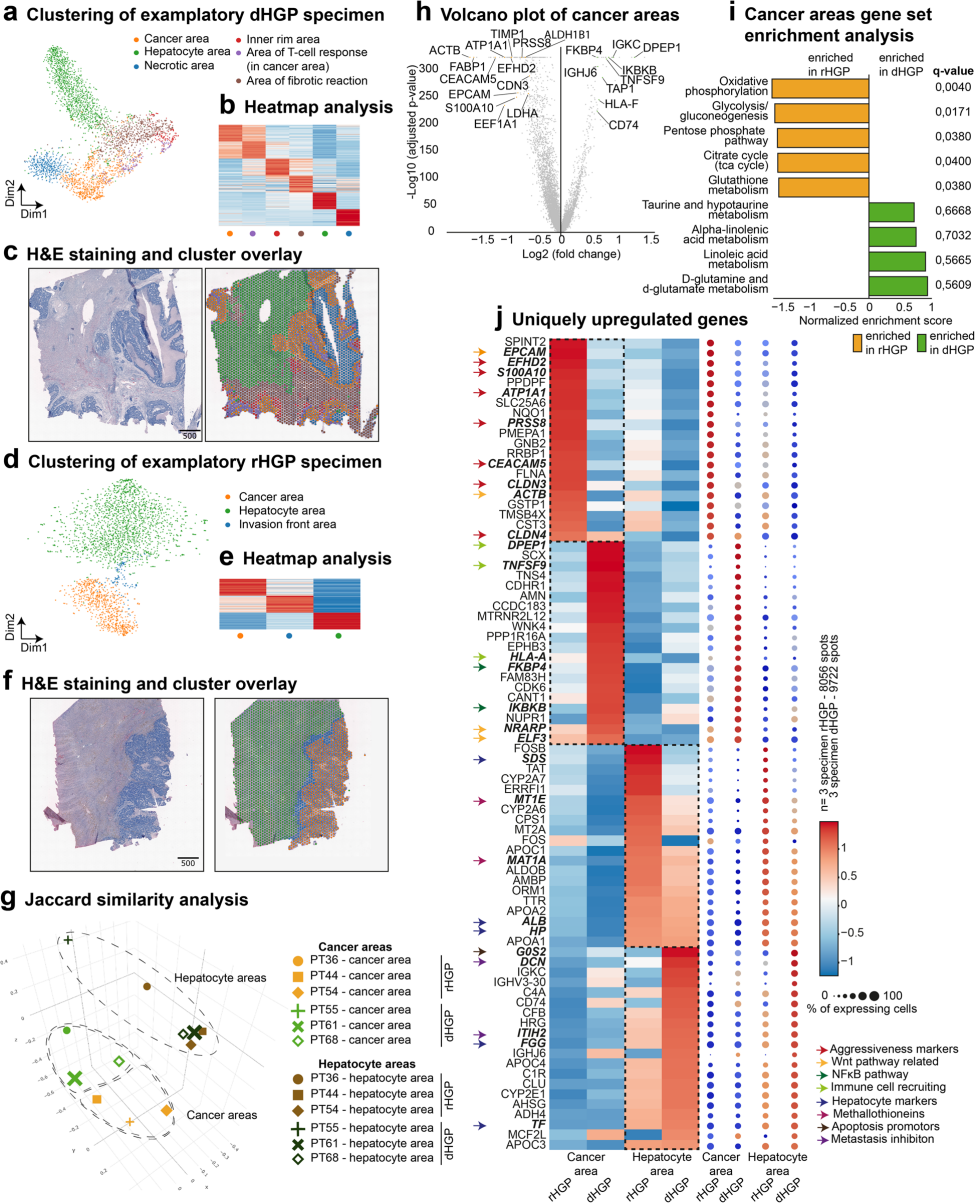
Spatial investigation of cancer areas reveals HGP-specific features. (**a**) representative t-SNE plot: spots of PT61 clustered via unsupervised Louvain clustering, biologically annotated. (**b**) Heatmap of 50 uniquely upregulated genes per cluster. (**c**) H&E staining of PT61 and overlayed Louvain-clustering. (**d**) representative t-SNE plot: 2,554 spots of PT54 clustered via unsupervised Louvain clustering, biologically annotated. (**e**) Heatmap of 50 uniquely upregulated genes per cluster. (**f**) H&E staining of PT54 and overlayed Louvain-clustering. (**g**) Jaccard similarity PCA on the pairwise Jaccard similarity coefficients between the marker genes of cells from cancer and hepatocytes area clusters in the samples from six different patients. (**h**) Volcano plot: DEA of cancer areas (dHGP vs rHGP), positive enrichment in dHGP. (**i**) Waterfall plot: GSEA comparing differentially expressed genes in cancer areas of dHGP vs cancer areas of rHGP (using KEGG metabolism, KEGG cellular processes as gene sets). (**j**) Heatmap and dot plot: top 20 uniquely upregulated marker genes of pooled spots for cancer areas and hepatic areas according to the HGP

First, we analysed the entirety of high-quality spots per sample using graph-based clustering, which was visualized via t-distributed stochastic neighbour embedding (t-SNE). The tissue type represented by each cluster was determined by analysing the top 50 marker genes and known canonical marker genes for the cell types present in every cluster (Supplementary Tables 4,5,6,7,8,9). Identification of tissue types was confirmed by overlaying clusters on images of the corresponding hematoxylin and eosin (H&E)-stained tissue. Clustering of the dHGP PT61 and the rHGP PT54 is depicted in Fig. 2a-f and the remaining patients are shown in Fig. S2a-h. When visualizing the entirety of spots from all patients together, we observed patient-wise clustering (Fig. S2i, j). Due to selection of the FFPE tissue slide, variable proportions of metastatic cancer areas versus healthy liver areas were identified in every sample (Fig. S2k). Finally, Jaccard similarity principal component analysis (PCA) comparing marker gene sets of cancer areas and hepatocyte areas of all patients showed that hepatocyte areas grouped separately from cancer areas, with additional HGP-wise subgrouping within the cancer areas (Fig. 2g).

To analyse HGP-specific features of cancer areas and hepatocyte areas, spots of all samples showing the same HGP and the same defined tissue type were combined. Differential expression analyses comparing cancer areas of rHGP and dHGP revealed established CRC cell markers, such as *FABP1, CEACAM5, CLDN3, EPCAM* and *S100A10* [28] in the rHGP (Supplementary Spreadsheet 1). Many of the detected marker genes are known to be involved in mechanisms enhancing the aggressiveness of cancer cells, such as epithelial-mesenchymal transition (EMT) (*EFHD2*, *ATP1A1* [29], *PRSS8* [30]), invasion (*S100A10* [30]), migration (*S100A10, EPCAM* [31]), poor prognosis (*TIMP1* [32]*, EEF1A1* [33]) and glycolysis (*ALDH1B1, LDHA*) (Fig. 2h). Interestingly, we also detected genes involved in WNT signalling activation. In detail, *EPCAM* is known to promote WNT signalling by stabilizing the WNT signalling co-receptor LRP6 and rescuing it from DKK2-induced removal from the membrane [34]. Furthermore, knockdown of *ACTB*, which was upregulated in our rHGP cancer areas, is known to decrease expression of β-Catenin, the main nuclear effector of canonical WNT signalling [35] (Fig. 2h). Moreover, among the enriched genes in the group of cancer areas in the dHGP, we detected many genes known to enhance inflammatory reactions in cancer by promoting the NFκB pathway (*IKBKB, FKBP4* [36]), activating lymphocytes (*TNFSF9, HLA-F*) [37, 38] or recruiting neutrophils (*DPEP1*) [39] (Fig. 2h). By dysplaying a gene set enrichment analysis (GSEA) for metabolic gene sets, we detected upregulation of genes involved in glycolysis/gluconeogenesis, the pentose phosphate pathway (PPP) and the antioxidative glutathione metabolism in rHGP cancer areas (Fig. 2i, Supplementary Spreadsheet 2).

Exploring enriched marker genes in cancer areas and hepatocyte areas of both patient groups, we observed hepatocyte areas identified by typical hepatocyte markers (*FGG, HP, SDS,)* of both HGPs to be rather congruent in comparison to cancer areas of both HGPs – a finding that was also confirmed by a marker gene intersection analysis visualized in dot plots (Fig. 2j, Fig. S2l-m and Supplementary Spreadsheet 3). Moreover, we detected genes coding for metallothioneins (*MT2A, MT1E*) uniquely upregulated in hepatocyte areas of rHGP CRCLM, possibly indicating a higher capacity in protection against ROS. Remarkably, hepatocyte areas adjacent to dHGP CRCLM showed upregulation of genes involved in tumor suppression and metastasis inhibition such as *G0S2, ITIH2 *[40] and *DCN *[41]. Interestingly, *DCN* overexpression in hepatocytes has been shown to attenuate especially aggressive phenotypes of CRCLM in vivo [42] (Fig. 2j, Supplementary Spreadsheet 4 and Supplementary Table 10).

In summary, our spaRNAseq results showed an increase of markers promoting tumor agressiveness and activation of WNT signalling in cancer areas of the rHGP, whilst markers of inflammatory reactions were upregulated in cancer areas of the dHGP. Strikingly, tumor suppressors were upregulated in hepatocyte areas adjacent to dHGP CRCLM that were detectable even though we observed high inter-patient heterogeneity (Fig. 2j).

### Upregulation of canonical WNT signalling in rHGP CRCLM

**Fig. 3:**
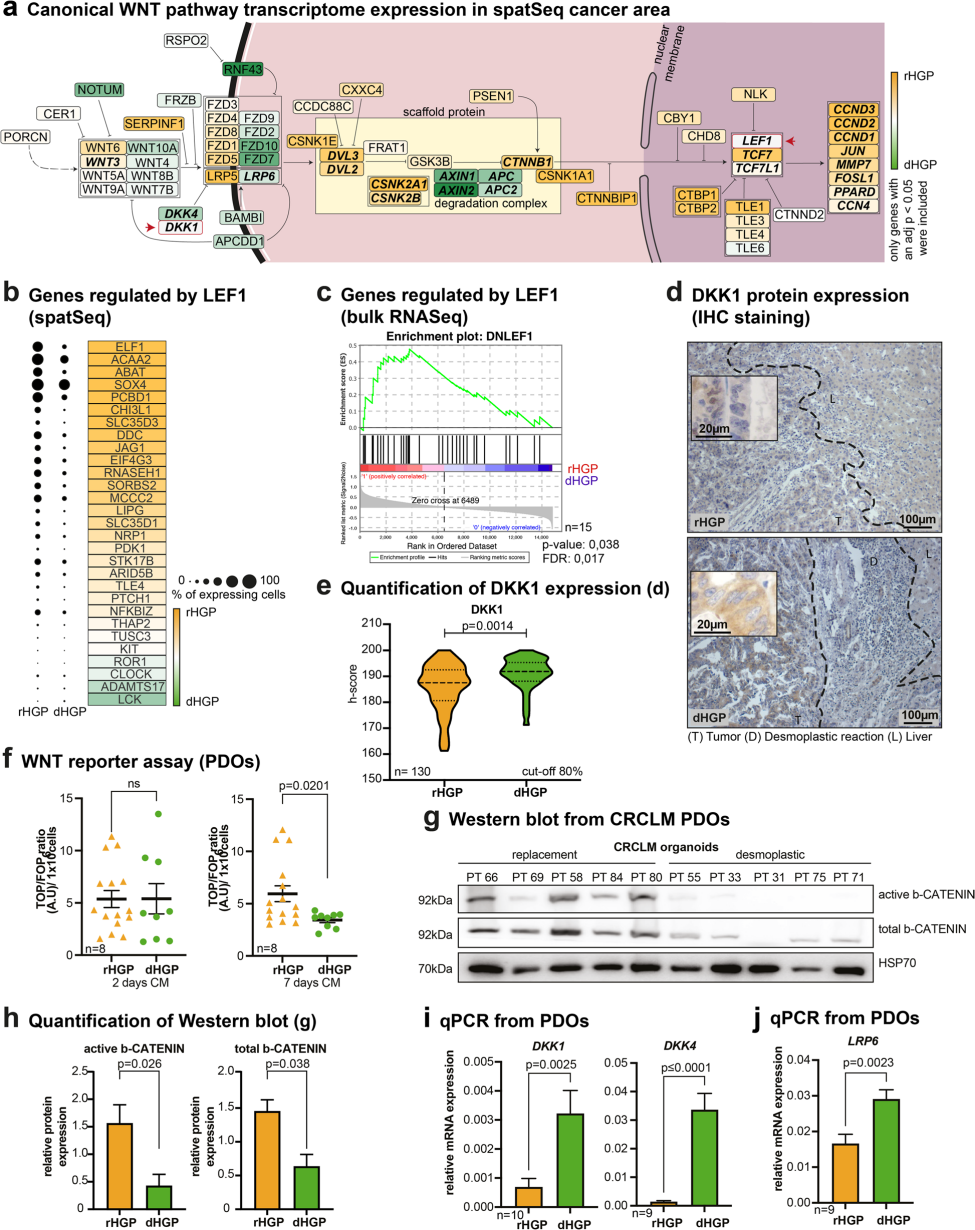
Upregulation of canonical WNT signaling in rHGP CRCLM. (**a**) Canonical WNT signaling pathway mapping: significant differentially expressed genes mapped according to the KEGG WNT signalling pathway. Colour coded according to scaled log fold change values. (**b**) Dot plot: significant differentially expressed genes regulated by LEF1. Mapped with expression percentages and colour coded according to scaled log fold change values. (**c**) GSEA: bulk sequencing data from CRCLM showing a gene signature regulated by LEF1 upregulated in rHGP (*n* = 6 rHGP vs *n* = 9 dHGP). (**d**) IHC staining: DKK1 (brown) in CRCLM (*n* = 130, cut-off 80% angiogenic or vessel-coopting HGP). (**e**) Violin plot: quantification of d. (**f**) Scatter plot: TOP/FOP-Flash WNT reporter assay with conditioned media from PDOs (*n* = 5 rHGP vs *n* = 3 dHGP). (**g**) Western blot: active and total ß-Catenin in protein lysates of PDOs (*n* = 5 rHGP vs n = 5 dHGP). (**h**) Western blot quantification of g. (**i**) qRT- PCR analysis for DKK1 (*n* = 6 rHGP vs *n* = 4 dHGP) and DKK4 (*n* = 4 rHGP vs *n* = 5 dHGP) in PDO-derived RNA. (**j**) Bar plot: RT-PCR analysis for LRP6 (*n* = 4 rHGP vs. *n* = 5 dHGP) in PDO-derived RNA. *P* values were calculated by unpaired t-test

Since we detected upregulation of several genes associated with WNT signalling activation in cancer areas of rHGP CRCLM, we further compared the activity of the canonical WNT signalling pathway in CRCLM of both HGPs. Using the differential gene expression analysis between cancer areas of both HGPs in our spaRNA-seq data, we mapped genes of the canonical WNT signalling pathway (Supplementary Spreadsheet 1). We observed an upregulation of *CTNNB1* and other genes involved in CTNNB1 stabilization (*CSNK2A1, CSNK2B, DVL2, DVL3,*) in rHGP cancer areas. In contrast, genes coding for major components of the β-Catenin destruction complex (*APC, APC2, AXIN1, AXIN2*) were upregulated in the dHGP. Furthermore, the majority of WNT signalling targets (*CCND3, CCND2, CCND1, JUN, MMP7, FOSL1, PPARD, CCN4*) were upregulated in the rHGP (Fig. 3a). Regarding secreted WNT signalling agonists and receptors, no clear trend in expression between the HGPs was observed, prompting us to explore the expression of WNT signalling antagonists. Among these, *DKK4* was upregulated in the dHGP (Fig. 3a).

*LEF1* is a key mediator of WNT signalling and is known to contribute to CRC progression and poor prognosis [43]. We therefore explored a signature of genes that were previously reported to be downregulated in CRC cell lines upon suppression of the canonical WNT signalling transcription factor *LEF1* (Supplementary Table 11). We detected that the majority of those genes are upregulated in cancer areas of the rHGP, thereby suggesting an expression signature associated with WNT signalling via *LEF1* (Fig. 3b).

For cross-data set validation, we extended our analysis to the only publicly available data set of bulk RNA-sequencing including information on HGPs CRCLM [19], which is composed of liver metastases tissue from six dHGP and nine rHGP CRCLM. Notably, GSEA revealed an enrichment of the *LEF1-*affected gene signature in the rHGP, corroborating our findings in the spaRNA-seq data set (Fig. 3c).

We next focused on analysing extracellular WNT antagonists since upstream WNT signalling mediators might possibly explain the differential regulation of WNT signalling between the HGPs. Therefore, we performed immunohistochemistry (IHC) on CRCLM FFPE sections for DKK1, the most extensively explored component of the DKK family of WNT signalling antagonists. Remarkably, DKK1 protein levels were found to be significantly increased in dHGP CRCLM (Fig. 3d-e).

We then investigated WNT ligand secretion using patient-derived organoids (PDOs) from CRCLM displaying either a predominantly rHGP or a dHGP (Supplementary Table 12). For this purpose, we collected CRCLM PDO supernatants after cultivation for two and seven days and performed a TOP/FOP-Flash WNT signalling reporter assay. Corroborating our previous findings (Fig. 3d-e), supernatants collected from rHGP CRCLM PDO supernatants after seven days showed an increased luciferase activity when compared to dHGP CRCLM PDO ones (Fig. 3f). These results strongly indicate increased WNT ligand secretion in rHGP CRCLM PDOs, which can lead to upregulated canonical WNT signalling in both autocrine and paracrine manners [44]. Strengthening this hypothesis, Western blot analysis performed with PDO lysates showed an increased expression of active and total β-Catenin in the rHGP PDOs (Fig. 3g-h). This finding was orthogonally confirmed by qRT-PCR analysis for *DKK1* and *DKK4* expression performed with RNA extracted from CRCLM PDOs with differing HGPs (Fig. 3i). Interestingly, *LRP6*, which is known to be upregulated by *DKK1* and downregulated by *WNT3A* [45], was also increased in PDOs RNA extracts from desmoplastic CRCLM (Fig. 3j) [45].

Taken together, these results reveal a higher activity of the canonical WNT signalling pathway in replacement CRCLM, whereas desmoplastic CRCLM express higher amounts of WNT signalling antagonists of the DKK protein family.

### Metabolic profiles of CRCLM according to their HGP

**Fig. 4:**
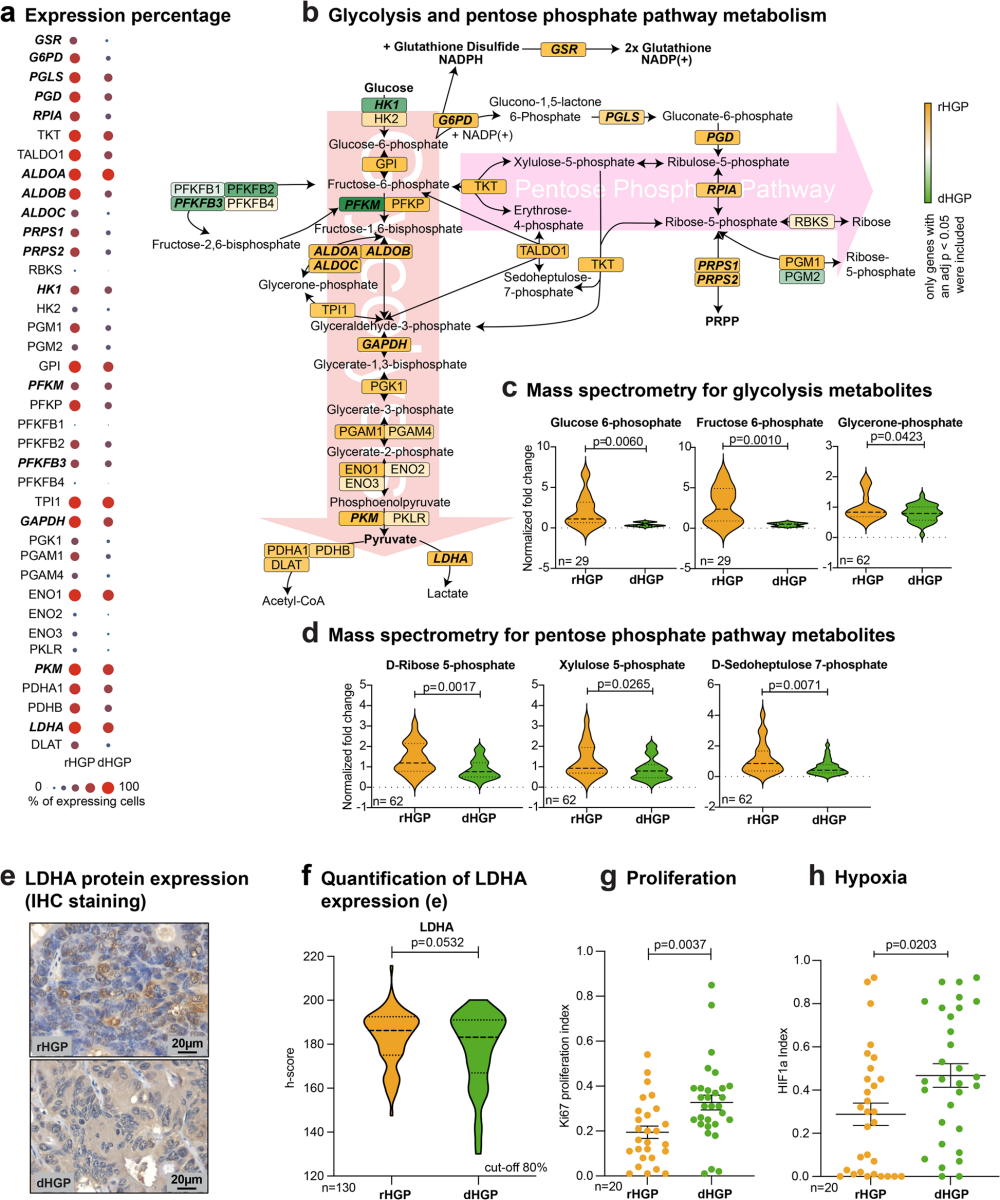
Metabolic profile of CRCLM. (**a**) Dot plot: significant differentially expressed genes involved in glycolysis and PPP mapped with expression percentages. (**b**) Pathway mapping: significant differentially expressed genes mapped according to the KEGG signaling pathway. Colour coded according to scaled log fold change values. (**c**) Violin plots: selected metabolites of glycolysis measured by mass spectrometry (*n* = 29 or *n* = 62). (**d**) Violin plots: selected metabolites of the pentose phosphate pathway measured by mass spectrometry (*n* = 62). (**e**) IHC staining: LDHA (brown) in CRCLM (*n* = 130, cut-off 80% angiogenic or vessel-coopting HGP). (**f**) Violin plot: quantification of e. (**g**) Scatter plot: quantification of IHC Ki67 staining (*n* = 10 rHGP vs *n* = 10 dHGP in triplicates). (**h**) Scatter plot: quantification of IHC HIF1a staining (*n* = 10 rHGP vs *n* = 10 dHGP in triplicates). *P* values were calculated by unpaired t-test

Since the WNT signalling pathway is known to upregulate aerobic glycolysis via the key enzymes pyruvate carboxylase and pyruvate dehydrogenase kinase [24, 46, 47], we further evaluated enzymes and metabolites of glycolysis and the branching PPP in both HGPs. Therefore, we mapped differentially expressed genes from the different cancer areas of the spaRNA-seq data involved in the respective pathways. Although some regulatory glycolytic enzymes, such as *HK1, PFKFB3, PFKM* were found upregulated in dHGP, the vast majority, including the key regulating enzyme PKM, was also upregulated in the rHGP CRCLM (e.g. *ALDOA, ALDOB, ALDOC, GAPDH, LDHA*) (Fig. 4a). As a branching pathway of glycolysis, we also detected upregulation of genes involved in the PPP in rHGP CRCLM (*PGLS, PGD, RPIA, PRPS1, PRPS2*). Furthermore, genes promoting cellular antioxidant defence (*G6PD*, *GSR*) were also upregulated in the rHGP CRCLM, thereby suggesting higher ROS scavenging capacity in the rHGP (Fig. 4b).

To further corroborate the unique metabolic identities of the CRCLM and to enhance the resolution of our molecular profiling to a single-cell level, we conducted scRNA-seq. Fresh tissue was derived from six different patients (Supplementary Table 13), of which three presented with the rHGP (PT44, PT54, PT59) and three showed the dHGP (PT52, PT55, PT61). Samples were taken from the metastases and healthy liver tissue. After quality control, 22,419 cells across all patients were included in the analyses (Supplementary Table 14).

Graph-based clustering, which we visualized via t-SNE, was used to group the entirety of cells. The cell-type identity represented by each cluster was determined by analysing the top 50 marker genes and known canonical marker genes for the cell types present in every cluster (Supplementary Table 15). Cell proportions, in relation to the total detected cells per patient, were calculated with immune cells being predominantly present within dHGP samples, whilst cancer cells, cholangiocytes, endothelial cells (ECs), fibroblasts and hepatocytes were predominantly detected in rHGP samples (Fig. S3a-f).

To verify metabolic molecular differences between cancer cells of the two differing HGPs, all cancer cells were subclustered. In total, 8803 cancer cells were included. Graph-based clustering, visualized via t-SNE, returned six independent clusters, two of which showed an activated phenotype expressing high levels of ribosomal genes. These clusters were mainly comprised from one patient each (Fig. S4a-e, Supplementary Table 16).

We next performed differential expression analysis (DEA) between cancer cells excluding the activated phenotype clusters since they masked the DEA due to high ribosomal gene counts (Supplementary Spreadsheet 5). We investigated changes between genes related to glycolysis and PPP, resulting in a similar expression pattern as in the spaRNA-seq data set, with both pathways upregulated in rHGP (Fig. S5a,b).

To validate these in silico findings, we performed mass spectrometry (MS) with fresh-frozen CRCLM bulk tissue from both HGPs. Again, we observed upstream metabolites of glycolysis (Glucose 6-phosphate, Fructose 6-phosphate, Glycerone-phosphate), which were significantly enriched in rHGP CRCLM (Fig. 4c). Moreover, metabolites of the PPP (Ribose 5-phosphate, D-Sedoheptulose 7-phosphate and Xylolose 5-phosphate) were also upregulated in rHGP CRCLM (Fig. 4d). Additional IHC analyses of CRCLMs from both HGPs confirmed upregulation of lactate dehydrogenase A (LDHA) in cancer cells of predominantly rHGP CRCLM (Fig. 4e,f).

Since the PPP is known to be upregulated during tumorigenesis, providing cells with structural nucleotide components [48], we explored the proliferative state of CRCLM in both HGPs by dysplaying IHC staining for Ki67. Unexpectedly, Ki67 was increased in dHGP CRCLM, suggesting that in rHGP, the PPP promotes cellular antioxidant defence rather than the supply of structural nucleotide components (Fig. 4g). Furthermore, proliferation as well as lower oxygen supply are potential drivers of glycolysis [49], which prompted us to additionally perform IHC staining for HIF1α, aiming to expose possible causes for enhanced glycolysis. In line with Ki67, HIF1α was found to be more strongly expressed in dHGP CRCLM samples (Fig. 4h), possibly explaining the larger areas of necrosis observed in dHGP CRCLM of our spaRNA-seq data as well as indicating a possible role in the induction of SA.

In conclusion, these results show that CRCLM patients displaying the rHGP exhibit enhanced glycolysis and PPP activation, which is associated with malignant progression and worse prognosis [50, 51]. Importantly, this intrinsic metabolic state seems to be independent of tumor cell proliferation or hypoxia.

### Phenotype of endolthelial cells detected in corresponding healthy liver tissue by scRNA-seq

After examining cancer areas and single cancer cells of CRCLM for their alterations in WNT signalling pathway and metabolic changes, we postulated that ECs of metastases with different blood supply mechanisms would likely express distinct transcriptomic signatures.

**Fig. 5:**
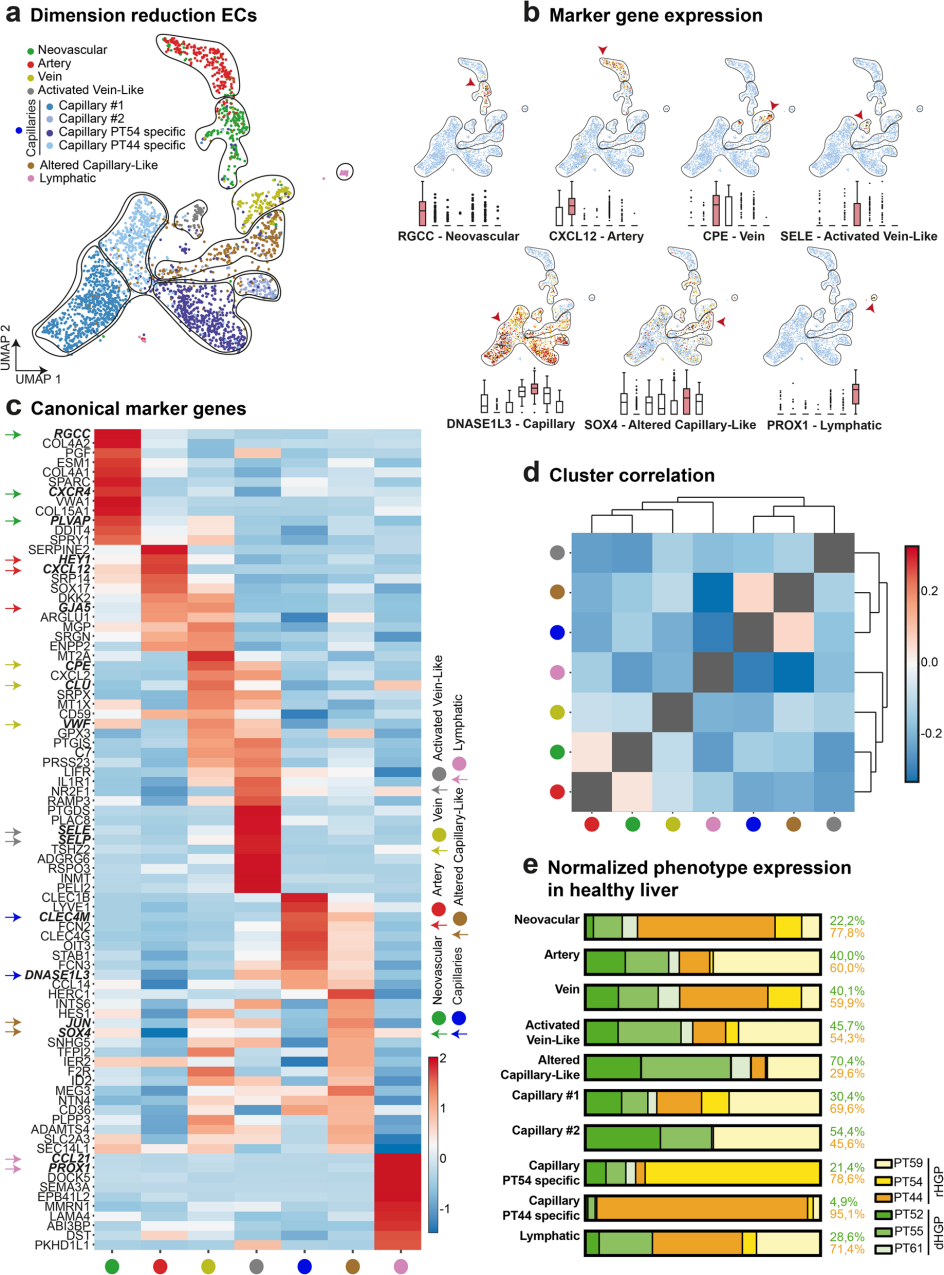
Specific capillary subtypes in corresponding healthy liver from patients with CRCLM. (**a**) UMAP plot: 2,654 analysed endothelial cells from healthy liver. Clusters identified via unsupervised Louvain clustering and biologically annotated. (**b**) Box plots and UMAP plots: quantification for each marker gene of the biologically annotated clusters. (**c**) Heatmap showing expression of canonical marker genes per cluster. (**d**) Correlation heatmap of annotated clusters. Hierarchical clustering location for row and column; confidence of branches estimated via bootstrapping (*p* = 0.05). (**e**) Bar plots: normalized cell amount and distribution of the origin of the analysed healthy liver endothelial cells

We therefore analysed a subset of 2,654 ECs collected from the corresponding healthy liver of six different CRCLM patients. Unsupervised Louvain-clustering, which we visualized via uniform manifold approximation and projection (UMAP), was used to cluster the ECs (Fig. 5a). The phenotype identification was determined by analysing the top 50 marker genes and known canonical marker genes for the cell types present in every cluster (Fig. 5b,c and Supplementary Table 17). Relevantly, the detected activated-capillary-like (ACL) cell population expressed canonical capillary marker genes, while also exhibiting a distinctly unique expression pattern on their own. Moreover, hepatic capillaries identified using previously described marker [52] do not express this additional set of genes (Fig. 5c). We next performed hierarchical clustering and bootstrap analysis to verify the identified clusters as distinct phenotypes (Fig. 5d). For biologically relevant clusters that were not represented by bootstrapping (capillaries and ACL), we confirmed statistical separation using DEA (Supplementary Spreadsheet 6). For each phenotype, a normalized expression percentage was calculated (Fig. 5e).

### Capillary heterogeneity in corresponding normal liver tissue of patients with different CRCLM HGPs

**Fig. 6:**
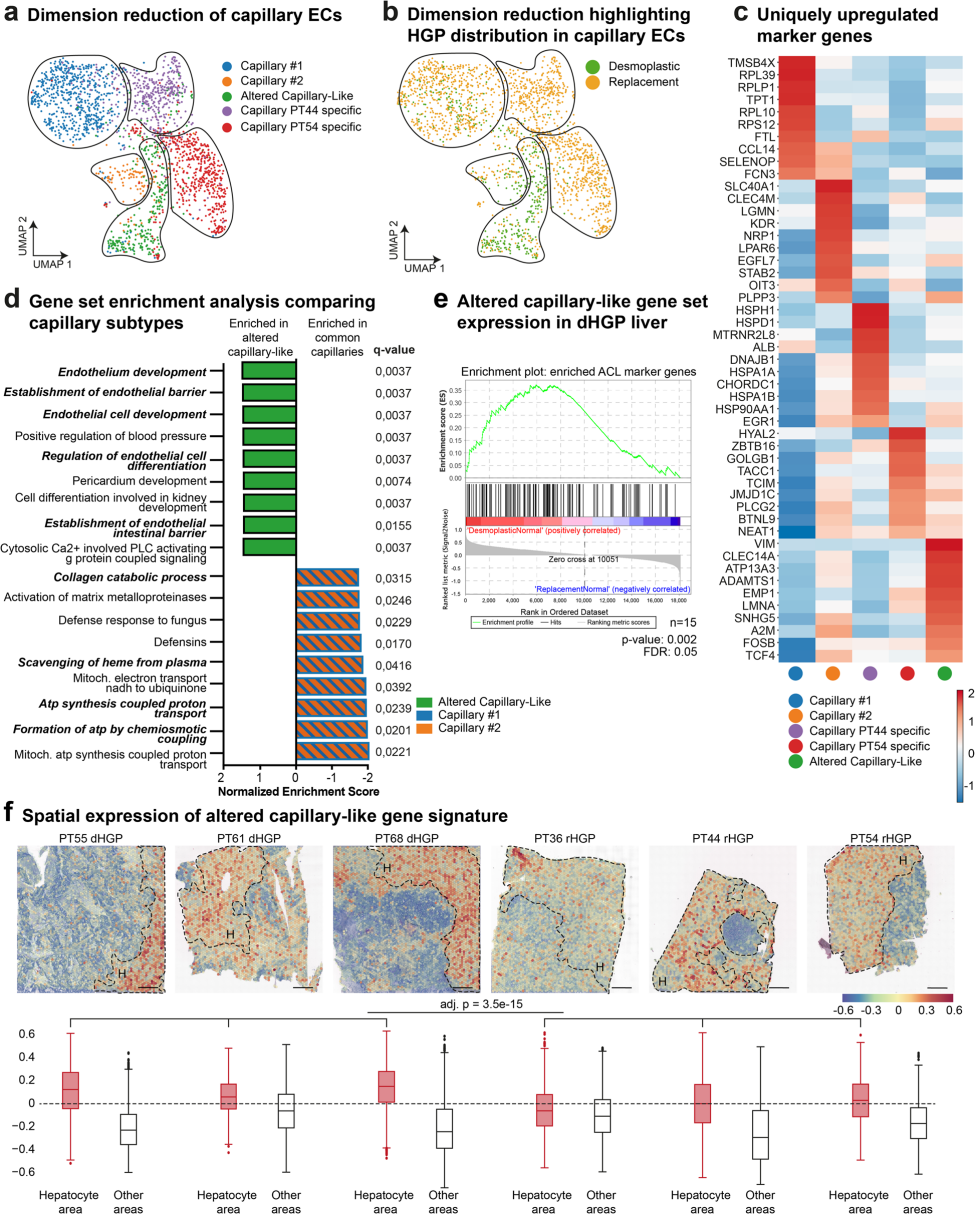
Capillary heterogeneity between HGPs in corresponding healthy liver from patients with CRCLM. (**a**) UMAP plot: 2,008 endothelial cells previously identified as capillary cells. Clustered via unsupervised Louvain clustering and biologically annotated. (**b**) UMAP plot: 2,008 analysed capillary cells. Colour coded for the HGP of sample of origin. (**c**) Heatmap showing 10 uniquely upregulated marker genes per cluster. (**d**) Waterfall plot: GSEA comparing capillary clusters 1 and 2 pooled vs ACL (using PID, KEGG, REACTOME, BP as gene sets). (**e)** GSEA: bulk sequencing data from corresponding healthy liver of CRCLM showing an upregulated signature of 150 marker genes of the ACL cluster in dHGP (*n* = 9 dHGP vs *n* = 6 rHGP). **(f)** Spatial overlay: spatial expression of a gene set of the 30 most enriched genes in ACL showing a relative enrichment in dHGP. Scale bars 500 µm

Subsequently, we conducted further analysis on these similar capillaries and ACL subgroups (Fig. 6a). Two clusters were predominantly formed by ECs collected from two single patients, whilst the two remaining capillary clusters were composed of cells from all six individual patient samples. The previously identified ACL cluster was mainly comprised of ECs derived from all three dHGP patients (Fig. 6b). Therefore, we performed marker gene analysis for enriched genes expressed for all capillary subclusters (Fig. 6c and Supplementary Table 18). Strikingly, the cytoskeletal filament vimentin was observed to be upregulated, when directly comparing all identified capillary subgroups. As vimentin is known to play a critical role in EC differentiation [53], cell adhesion and endothelial sprouting [54], we assumed that the ACL cells engage in processes that support the formation of functional vessels and endothelial barrier integrity needed for vascular homeostasis and vessel formation.

To better understand the involvement of vimentin in the context of cellular pathways, we performed DEA and GSEA comparing canonical capillaries to the new described ACL (Fig. 6d and Supplementary Spreadsheet 6). Among the significant and most upregulated pathways in ACL cells, the MSigDB pathways “establishment of endothelial barrier”, “regulation of EC differentiation” and “EC development” were top ranked (Supplementary Table 19). Within the included genes of these specific gene sets, *NOTCH4* and *CTNNB1* appear within the most upregulated ones, indicating that this novel phenotype indeed has an underlying expression of genes canonically known to be involved in regulating SA (*NOTCH4*) [55, 56] and EC barrier function (*CTNNB1*) [57] (Supplementary Spreadsheet 6). Additionally, closer examination of the upmost regulated genes (*Vimentin, CLEC14A, ADAMTS1* and *EMP1*) and their available literature shows a interconnection with angiogenic processes. Vimentin, a cytoskeletal filament well-known for its regulation on cell shape, migration and invasion [58], and a hallmark for epithelial-mesenquimal transition (EMT) has important roles in angiogenesis [59–62]. CLEC14A, a regulator of sprouting angiogenesis is considered a tumor endothelial marker, and its blocking has shown to decrease vascular density and the ability of sprouting angiogenesis [63]. Murine haploinsuficiency of ADAMTS1, a desintegrin and metallopeptidase with described angiogenic-related functions causes thoracic aortic aneurysms and dissections similar to Marfan syndrome [64], while silencing of the poorly investigated small hydrophobic membrane-associated protein EMP1 inhibis cancer cell proliferation, migration, and affects VEGF-C expression in nasopharyngeal cancer, reducing angiogenesis [65, 66].

As we identified a neovascular cluster expressing canonical marker genes (Fig. 5c), we interpret this finding as a novel capillary subgroup with assumed heterogeneity between the different HGPs. Thus, suggesting that independent from the classical SA phenotype, the normal hepatic parenchyma appears to host a microenvironment that possibly promotes vessel sprouting. Addtionally, dysplaying cross-data set validation, we confirmed the enrichment of the ACL phenotype in healthy dHGP liver by dysplaying GSEA on independent bulk sequencing data from rHGP and dHGP [19] using the enriched marker genes of the ACL cluster (Fig. 6e, Supplementary Table 20).

Finally, we investigated the spatial distribution of this phenotype by constructing a gene set containing the 30 most enriched marker genes from the ACL phenotype data set (Supplementary Table 20) and then displayed it on the spaRNAseq data set. We confirmed the presence of this gene set in the corresponding healthy liver tissue with a relative enrichment of these capillaries in dHGP. Remarkably, this signature was absent in the tumor tissue (Fig. 6f).

## Discussion

rHGP CRCLM dysplaying VCO are characterized by a worse prognosis and by an impaired response to anti-angiogenic treatment when compared to dHGP CRCLM dysplaying SA [4, 67, 68]. Since rHGP CRCLM require a wider surgical margin, a liquid biomarker diagnostic tool to determine the HGP of CRCLM without pathological work-up would be important not only for systemic therapy decisions, but also for surgical strategies [69]. Thus, to identify new possible therapeutic targets for biomarker candidates in general and rHGP CRCLM in particular, we characterized differing features of the main HGPs in the present study.

We validated the observation of improved OS in *pure* dHGP CRCLM [4, 70]. Thus, the *pure* dHGP can be considered as a positive prognostic marker, whereas any amount of non-desmoplastic HGP is associated with impaired survival rates. The beneficial OS for dHGP in general and in a pre-treatment-naive subgroup of patients in particular along with the loss of this benefit in the pre-treated subgroup is in line with the results obtained by Galjart et al. [4]. Interestingly, liver metastases derived from other primary tumors than CRC also display HGPs that are morphologically similar to the ones described for CRCLM and seem to have a comparable prognostic value [71, 72]. Taken together, these findings underscore the prognostic relevance of the HGP.

In CRCLM, anti-RAAS medication is associated with an improved response to anti-angiogenic therapy through reduced mechanical activity of the metastases-associated fibroblasts [14]. Considering the different composition of the stroma in CRCLM with different HGPs [9, 10], we performed survival analyses considering the HGP and the application of anti-RAAS medication. Interestingly, the application of anti-RAAS medication is associated with a trend for improved OS in patients with *pure* dHGP, which can be explained by the association of RAAS activity in the promotion of SA [42, 73, 74], a prominent feature of dHGP CRCLM. Importantly, we observed that anti-RAAS medication was associated with improved survival in the subgroup of pre-treatment-naive patients with *pure* dHGP.

Additionally, we observed a higher occurrence of the rHGP in CRCLM originating from primary rectal tumors as well as a tendency towards exhibition of the dHGP in CRCLM originating from the cecum and colon ascendens primary tumors. Previous studies have already characterized prognostic and molecular differences between right- and left-sided CRC primary tumors, such as increased microsatellite instability in right-sided tumors and more frequently occurring *APC and TP53* mutations in left-sided tumors. To add to this characterization, this study has revealed that right-sided primary tumors are associated with CRCLM displaying sprouting angiogenesis, whereas primary tumors originating from the sigmoid and rectum tend to develop CRCLM with VCO.

A high concordance between CRC primary tumors and their matched liver metastases has been reported on a genetic and mutational level, even if they differ in regard to their transcriptome and proteome [75, 76]. Comparing the mutational status in right- and left-sided CRC primary tumors also revealed that location-associated differences in APC mutations have an impact on the fine-tuning of canonical WNT signalling in CRC primary tumors. These differences are in parallel with the WNT signalling differences that we have observed in the two main HGPs of CRCLM when considering their origin from proximal or distal colon in our single-centre cohort [77].

In our spaRNA-seq data set, we observed activation of the WNT signalling pathway in different HGPs. As one of the most frequently mutated pathways in CRC, aberrant WNT signalling in both tumor cells and in the TME has an impact on carcinogenesis, tumor growth, metastasis and OS [78]. Our findings of DKK1 expression enhancement in dHGP CRCLM could shed light on the biological mechanisms dictating the differences between growth patterns. In this context, it was recently shown that WNT antagonists released by CRC organoids could trigger strong desmoplastic reactions in vivo [79], and therefore, promote the fibrotic reaction observed in dHGP. Further in vivo CRCLM setups, using DKK1-overexpressing cells could deeper our understanding in this process. Moreover, our WNT signalling findings are in line with recently proposed theories for specific metastatic histological features and growth patterns such as “histostasis” [80] and “histokinesis” [6].

Indeed, dHGP CRCLM resemble primary CRCs from a morphological point of view (‘histostasis’) since this type of CRCLM has differentiated, crypt-like structures that are also present in most primary CRCs. These crypt-like structures resemble crypts of the normal mucosa of the large intestine, suggesting that inhibition of WNT signalling plays a role in the establishment of these structures [81]. In rHGP CRCLM, cancer cells at the tumor-liver interface are arranged in solid nests, thereby not mirroring the morphology of primary CRCs (‘histokinesis’). Thus, the results of the current study showing increased WNT signalling related to rHGP could, at least in part, explain the lack of crypt-like structures in rHGP CRCLM.

Importantly, WNT signalling has already been shown to promote VCO in other cancer entities. In detail, WNT7A/B secretion by Olig2 + oligodendrocyte precursor-like glioma cells was observed to promote VCO and to be upregulated in response to anti-angiogenic treatment [82]. Furthermore, WNT7B was highly expressed in invasive cancer cells dysplaying VCO in a renal cancer lung metastasis mouse model [83], indicating a role of WNT signalling in VCO across different cancer entities.

Of interest is that we observed that cancer cells from the rHGP display an intrinsic strong glycolytic profile, which is strongly associated with a more aggressive phenotype [50, 51]. This observation is further supported by a previous report that a higher baseline glucose uptake of CRCLM was detected in non‐dHGP patients, when compared with dHGP patients [84]. Accordingly, the rHGP is associated with the activation of the PPP, which plays a role in antioxidant defence, thereby suggesting higher resistance to ROS in rHGP CRCLM. Since ROS can induce cancer cell death [85], higher ROS resistance could lead to survival benefits of cancer cells, and thereby, contribute to the more aggressive phenotype of rHGP CRCLM.

To compare the different HGPs of CRCLM, a previous study has only conducted bulk RNA sequencing analyses [86] rather than a detailed analysis of different involved cell types at a single-cell level. To directly fill this gap, we performed scRNA-seq from CRCLM paired with their corresponding macroscopically healthy liver tissue. On the transcriptomic level, we observed a type of capillary-like ECs enriched in healthy liver tissue derived from dHGP CRCLMs, compared to healthy livers showing rHGP CRCLM. Those capillary-like ECs showed upregulation of gene signatures for EC and vascular formation. This finding could be of crucial diagnostic interest in predicting the HGP since hepatic biopsies containing healthy liver regions could be further analysed in regard to this EC’s transcriptional signature.

Finally, our study provides a publicly available tool for data exploration through the Unicle webtool (https://unicle.life/portals/) to ensure data accessibility to non-bioinformaticians, analysis reproducibility and resource value. In conclusion, our findings suggest that glycolysis, the WNT signalling pathway and the evaluation of capillary-like ECs signatures could be further exploited as possible targets for the treatment of rHGP CRCLM.

## Conclusions

In this study we have detected specific metabolic alterations and a signature of WNT signalling activation in metastatic cancer cells related to the VCO phenotype. Importantly, in the corresponding healthy liver of CRCLM displaying sprouting angiogenesis, we identified a predominantly expressed capillary subtype of endothelial cells, which could be further explored as a possible predictor for HGP relying on sprouting angiogenesis. Together, our data shed light on new therapeutical targets in CRCLM relying on VCO.

## Supplementary Information

**Additional file 1.** 

**Additional file 2.**

## Data Availability

All spaRNA-seq and scRNA-seq data, (including accession codes), are available in the ArrayExpress database [87], (http://www.ebi.ac.uk/arrayexpress) under accession number E-MTAB-12022 (scRNA-seq) and E-MTAB-12043 (spaRNAseq). To ensure data accessibility to non-bioinformaticians, reproducibility, and resource value, we made our scRNA-seq data available for further exploration via an interactive webtool: https://unicle.com/portals/. Using this tool, users can interactively visualize gene expression and clustering on t-SNE, search marker genes for all subclusters and export gene expression data. Bulk mRNA-seq expression data (GSE151165 [19]) were downloaded from the Gene Expression Omnibus (GEO) database (https://www.ncbi.nlm.nih.gov/geo/).

## Abbreviations

CRC: Colorectal cancer
CRCLM: Colorectal cancer liver metastasis
HGP: Histological growth pattern
rHGP: Replacement histological growth pattern
dHGP: Desmoplastic histological growth pattern
pHGP: Pushing histologica growth pattern
VCO: Vessel co-option
SA: Sprouting angiogenesis
EC: Endothelial cells
scRNA-seq: Single-cell RNA sequencing
spaRNA-seq: Spatial RNA sequencing
RAAS: Renin–angiotensin–aldosterone system
FFPE: Formalin-fixed-paraffin-embedded
t-SNE: t-distributed stochastic neighbour embedding
PCA: Principal component analysis
PPP: Pentose phosphate pathway
IHC: Immunohistochemistry
H&E: Hematoxylin and Eosin
UMAP: Uniform manifold approximation and projection
EMT: Epithelial-to-mesenchymal transition
ACL: Activated-capilary-like
DEA: Differential expression analysis
MS: Mass spectrometry
GSEA: Gene set enrichment analysis
MSigDB: Molecular signature database
PID: Pathway Interaction database
KEEG: Kyoto enciclopedia of genes and genomes
BP: Gene ontology “biological process”
OS: Overall survival
HIF1: Hypoxia-inducible factor-1
VEGF: Vascular endothelial growth factor
LDHA: Lactate dehydrogenase A
TME: Tumor microenvironment
ROS: Reactive oxygen species
