# Supplementary material for: Molecular differences of angiogenic versus vessel co-opting colorectal cancer liver metastases at single-cell resolution
Source: Mol Cancer. 2023 Jan 24;22:17. doi: 10.1186/s12943-023-01713-1 (PMC9872436; doi:10.1186/s12943-023-01713-1)
Supplement: Supplementary file 1 — Additional file 1. [file 12943_2023_1713_MOESM1_ESM.pdf]

Supplementary Table 1a-b. Related to Figure 1 and Figure S1

a. Clinical Characteristics of 225 patients resected for CRCLM

		Patients (No.)
**Total (No.)**		225
**Sex (No.)**	Female	84
	Male	141
**Age at time of CRCLM resection (Years)**	Mean	63,04
	Min	33
	Max	84
**initial postoperative UICC (No.)**	I	13
	II	42
	III	50
	IV	117
**Localization of the primary tumor (No.)**	Coecum to Colon ascendens	24
	Right colic flexure to Colon descendens + Sigma	68
	Rectum	133

Note:

Patients were resected for CRCLM in a timeframe of 1995-2015; Initial postoperative UICC describes UICC at timepoint of resection of primary tumor; Neoadjuvant treatment: within 6 months prior to CRCLM resection

b. Multivariate analyisis from CRCLM patients in cohorts Figure 1c-d

Variables		Hazard Ratio (HR)	*P* value
**dHGP**	*Unpure* - 100%	0.4266571	0.0002819
**pUICC**			
	I -		
	II	1.0762858	0.8669213
	III	1.4598015	0.3730300
	IV	1.8509338	0.1266244
**Age**			
	<= 60 -		
	age 61-70	0.9764811	0.9018906
	age>70	1.5242961	0.0508221
**Gender**			
	F -		
	M	0.7618939	0.1137023
**Localization**			
	Rectum -		
	Right colic flexure to Colon descenens + sigma	0.9574421	0.8142765
	Coecum to Colon ascendens	1.8482461	0.0211604

Multivariate Cox Proportional Hazards Analysis for Overall Survival, performed with the variables dHGP (=100%), pUICC, age, gender and localization

Supplementary Table 2. Related to Figure 2.

Clinical Characteristics of 6 patients resected for CRCLM used for spaRNASeq

PT-ID	HGP	Gender	Age at resection	Year of resection	Initial postop. UICC	Location primary tumor	MS status	KRAS mutation	RAS inhibition	Pre-treatment
PT36	rHGP	Male	68	2020	IV	Rectum	MSS	yes	No	FOLFIRI, Bevacizumab
PT44	rHGP	Female	69	2020	III	Colon asc.	MSS	No	No	No
PT54	rHGP	Female	53	2020	IV	Rectum	MSS	No	No	FOLFOX
PT55	dHGP	Female	67	2020	IV	Rectum	MSS	Gly12Val	Candesartan	No
PT68	dHGP	Female	62	2021	I	Rectum	MSS	No	No	5-FU/FA, FOLFOX
PT61	dHGP	Male	77	2020	IV	Sigma	MSS	No	No	No

Supplementary Table 3. Related to Figure 2

Quality metrics spaRNASeq

patient ID [a]	HGP [b]	number of reads [c]	number of spots under tissue [d]	UMIs/spot [e]	genes/spot [f]	saturation [g]	number of spots under tissue after exclusion	reason for exclusion[h]
PT55	dHGP	191032701	4924	5925	2926	77,7%	4857	1
PT68	dHGP	155654697	4940	14008	4042	31,6%	4226	1;2
PT61	dHGP	231767542	3434	5141	2630	80,3%	3434	/
PT36	rHGP	141418423	3890	6768	3334	48,6%	3890	/
PT44	rHGP	185134567	2483	22948	5583	53,7%	2843	/
PT54	rHGP	164424295	2601	6043	2838	73,5%	2554	3

Notes:

a *the patient identifier of each sample*

b *specifies whether the sample was derived from desmoplastic or replacement CRCLM*

c *the total number of reads per sample*

d *the number of spots under tissue per sample (according to spaceranger count)*

e *the median number of UMIs detected per tissue covered spot prior to exclusion*

f *the median number of genes detected per tissue covered spot prior to exclusion*

g *sequencing saturation equals the fraction of reads originating from an already-observed UMI*

h *reasons for exclusion: 1:gene count below the threshhold of 200 genes/spot; 2:ambiguous expression of canonical marker genes; 3:folded tissue on slide*

Supplementary Table 4. Related to Figure 2

Uniquely upregulated genes of PT61 for each biologically annotated cluster (spaRNASeq)

Rank	Cancer area	Hepatocyte area	Fibroblastic reaction	Necrotic area	Mainly T-cells within TU	Inner rim
1	PHGR1	FGG	PTGDS	SERF1A	ATP5MC3	IGKC
2	PRR15L	ALB	TPM2	SERF1B	HLA-DPB1	IGHG1
3	MAP7	FGA	PPP1R14A	MTRNR2L12	MIF	IGHG3
4	ID4	HP	MYH11	FAM153B	HNRNPD	IGHV3-30
5	MACROD1	TF	TMEM119	UPK3BL1	HLA-DRA	IGHJ6
6	HSPD1	AHSG	MOXD1	POLR2J3	MUC6	LYZ
7	PLA2G2A	APOA1	ELN	SPACA7	TAP2	TIMP1
8	DDR1	RBP4	APOBEC3F	USP17L19	TMSB4X	IGKV3OR2-268
9	PA2G4	HPX	FEZ1	TAF11L3	PSMB9	IGKV1D-12
10	NOP53	CLU	IGHV1-46	USP17L23	HLA-DPA1	COL1A2
11	HNRNPA3	APOC3	GLT8D2	USP17L8	MAPKAPK2	C1QC
12	EBPL	VTN	ESR2	TAF11L2	NDUFA11	IGLC2
13	CCT7	CRP	C1QTNF7	MYO16	TSPO	SPARC
14	WNK4	FGB	STMN2	CA9	NAPRT	TYROBP
15	DDT	C3	NUAK1	USP17L5	COTL1	LAPTM5
16	EPCAM	MT1X	PPP1R3F	POTEH	TONSL	IFI6
17	ASCL2	G0S2	SLC2A5	USP17L18	SOX9	IGHV3-35
18	EPHB2	HRG	GPBAR1	USP17L15	IFITM3	FN1
19	BSPRY	CYP2E1	CD1E	USP17L3	RHPN1	IGFBP5
20	SFN	C1R	IGHD	TAF11L6	SSR1	FLNA
21	KCNQ1	C4A	CPT1C	FAM72A	CYBA	COL3A1
22	MCM7	ITIH2	SUSD5	TAF11L9	NDUFA3	IGFBP7
23	AXIN2	A1BG	PPOX	SPDYE5	UBE2S	TYMP
24	SAPCD2	APOB	VPS50	GOLGA8N	UQCR11	COL1A1
25	TSPAN8	AZGP1	VXN	CBWD5	TAF15	IGHV3OR16-13
26	PRR13	TTR	FGF13	NBPF12	SNRPC	IGHG2
27	MAPK13	POR	KCNT2	TAF11L13	INTS1	COL4A1
28	TUBA4A	APOA2	TAFA4	USP17L13	INO80B	VIM
29	DNAJB1	SERPINA1	DACT3	ANKRD20A1	SNRPD3	ITGB2
30	HSPE1	FABP1	CASD1	USP17L10	SNRPF	F2R
31	REPIN1	CYP4A11	ZNF773	ZNF706	PAPOLA	CTSB
32	CAMK2N1	ORM1	HORMAD1	USP17L1	INCENP	C1QB
33	CDC42EP5	ALDOB	MYL4	POTEG	MLLT6	CAVIN1
34	EPHB4	MT2A	LRAT	IFNA4	GPAA1	PSAP
35	CDK4	ASGR1	FGF8	TAF11L8	NDUFS8	S100A4
36	BRI3BP	RASD1	SMUG1	PPIAL4D	HNRNPA2B1	IGKV4-1
37	CYP2S1	TFR2	PCDHB5	NDRG1	DYNLL2	CCN2
38	PRRC2A	FBP1	VSIG2	BAALC	ZGPAT	EMILIN1
39	PLEKHG5	IGFBP1	CCR4	LIG4	ZMIZ2	IGLC3
40	EIF6	HPD	ZNF594	CBWD6	EPPK1	LSP1
41	HES1	CFB	PODN	GOLGA8F	ARHGEF1	CTSD
42	MLEC	HMGCS2	IFNL1	TRIM64B	HSD17B10	CTSZ
43	HSP90AA1	APOE	DIRAS3	AGAP9	SLC52A2	CAPG
44	DNPH1	CYP3A5	SOX15	FSBP	PDAP1	CCL18
45	DACH1	ADH4	ANKEF1	AMY2B	TMEM160	GSN
46	ERGIC3	APOC1	ZNF223	ABHD13	RUVBL2	C11orf96
47	SNHG32	GOLPH3	UCP3	FAM72D	MUC5AC	IGFBP4
48	TAF10	APOC2	TMEM132E	TCEA1	IFI27	HTRA3
49	RAB25	FGF21	C14orf28	SNRPG	MYBL2	CD74
50	IMPDH2	DCN	OPRL1	SYNE1	SF3B5	CTSC

Supplementary Table 5. Related to Figure S2

Uniquely upregulated genes of PT68 for each biologically annotated cluster (spaRNASeq)

Rank	Hepatocyte area	CAFs/Macrophages (in Cancer area)	T- /B-cell infiltration(in hepatic area)	Inner rim	Necrotic area	Cancer area
1	APOC3	SULF1	SAA2	UBD	SERF1A	PHGR1
2	ALB	COL1A1	CRP	TNC	SERF1B	OLFM4
3	TF	MMP9	SAA1	CDKN2A	USP17L5	EPCAM
4	TTR	MMP11	C3	TAP1	USP17L19	LGALS4
5	AHSG	VIM	FGA	CYCS	TAS2R20	CEACAM5
6	HRG	SPP1	MT2A	TGM2	MYOZ1	ELF3
7	ADH4	AEBP1	LBP	S100A14	OR4F21	KLF5
8	APOA1	COL1A2	G0S2	RAN	TAF11L3	CDH17
9	CYP2E1	CTSB	IL32	RNF213	IGF2BP3	SPINT2
10	APOA2	POSTN	SERPINA3	TAP2	PFDN4	NOP53
11	RBP4	ACTA2	SERPINA1	ARGLU1	EPYC	PPA1
12	CLU	TAGLN	CFB	RAB5IF	C17orf107	CLDN3
13	ASS1	MFGE8	C4B	EPSTI1	TAF11L2	TFF3
14	ALDOB	LAPTM5	DCN	ARPC1B	C19orf85	CD9
15	AMBP	CTSD	MT1M	EIF5A	RXFP2	S100A10
16	ADH1B	CCN2	C1R	BCL2L1	OR4F4	PPP1R1B
17	CYP2A6	LUM	LTB	ILF3	SLC5A7	CLDN7
18	APOC1	FLNA	MT1B	TRIM31	OR2AK2	GOLM1
19	HP	IGFBP5	CORO1A	HDGF	USP29	PLCB4
20	FGG	CST1	CXCL12	CLTB	OR4K15	HMGN1
21	GC	SPARC	NNMT	NFKB2	PRAMEF17	LSR
22	GSTA1	PLAUR	MT1X	OAS3	KLRF2	FKBP4
23	AZGP1	LYZ	SPON2	PLSCR1	SNAP91	YWHAZ
24	CYP2A7	INHBA	FGL1	ACTG1	MB	KRT8
25	VTN	COL4A1	ITIH3	CDK6	OR4K13	LSM7
26	ITIH2	COL3A1	LRG1	COTL1	RAD21L1	HMGB2
27	APOB	GSN	PLA2G2A	GATAD2A	DGKZ	MCM7
28	APOC4	COL6A3	C1S	TAGLN2	MPP4	PRSS8
29	FGB	GREM1	MT1E	ARPC2	GLP1R	CLIC1
30	KNG1	GPNMB	SOD2	EIF4A1	USP17L15	UBE2C
31	MAT1A	CD68	CNDP2	SRRT	OR2T27	SH3BGRL2
32	CYP4A11	SOD3	GLUL	JPT1	LRRD1	TPI1
33	APOH	VCAN	SLC39A14	CENPW	CEACAM21	HSP90AA1
34	ORM2	CAPG	TNXB	HNRNPUL1	MS4A13	HSPA1B
35	HPD	MMP14	HLA-DMA	LAMC2	ANKRD20A1	CDH1
36	HPX	THBS2	COL6A1	FLII	SPDYE5	PLEKHG6
37	AFM	IGFBP7	NFIC	CDC37	FAM163A	TSPAN8
38	CYP2B6	TPM2	GOLPH3	LMO7	FCRL2	FAM3D
39	AGT	HLA-B	SEPTIN1	HLA-C	USP17L18	CHMP4B
40	CYP2C8	FN1	IKZF1	UBE2M	UBQLNL	MDH2
41	F2	MMP12	PTGDS	NME1	CASQ1	EIF6
42	TTC36	TIMP3	STOM	HNRNPM	TAS1R2	MIF
43	A1BG	PSAP	BST2	PTGES3	TSSK3	SDC4
44	HAMP	CD74	TNFSF14	GTF3C6	HPGDS	RPIA
45	PLG	RNASE1	CIITA	CDC25B	GPR18	SLC25A6
46	ITIH1	IFI6	KLHL5	DDX21	LYZL6	PSMA7
47	ADH1A	NUPR1	TRBC1	VASH1	LRRC66	TPX2
48	PCK1	B2M	PHYKPL	NUTF2	CASP5	PFKFB2
49	CPS1	PMEPA1	APOL1	SNRPD1	SPAM1	LDHB
50	SLC27A5	HLA-DRA	SKAP1	RASSF4	CLRN2	SRSF3

Supplementary Table 6. Related to Figure S2

Uniquely upregulated genes of PT55 for each biologically annotated cluster (spaRNASeq)

Rank	Hepatocyte area	Cancer area	Necrotic area	Fibrosis + Infiltration
1	CLU	PIGR	SERF1A	IGHJ6
2	A1BG	PPP1R1B	SERF1B	IGKC
3	FGB	SCRIB	MTRNR2L12	IGHV3-30
4	VTN	TSPAN8	USP17L19	COL1A2
5	CFB	CDHR1	USP17L18	COL3A1
6	TF	MUC2	TAF11L2	IGHV3-35
7	CYP2E1	DDR1	USP17L3	IGLC2
8	RBP4	COX6C	USP17L8	TPM2
9	HP	CKB	USP17L5	IGHV3OR16-13
10	AHSG	FAM3D	TAF11L8	COL1A1
11	MT1G	PHGR1	TAF11L9	TIMP1
12	CRP	CLDN7	CT45A10	AKR1B1
13	C3	EPHB3	POLR2J3	SPARC
14	ALB	CEACAM5	TAF11L3	IGFBP7
15	SAA2	AMN	RNF19B	LAPTM5
16	HPX	HSF4	RNASEL	PDGFRB
17	SERPINA1	CYP2S1	USP17L15	CIITA
18	FGA	SPNS2	SLC66A3	FLNA
19	APOA1	CEP170B	TAF11L13	PLXND1
20	ITIH2	MYH14	KCNK6	FBN1
21	FGG	CCDC183	RNF20	HLA-DOA
22	IGFBP1	SPINT2	ZNF527	IGHG1
23	C4A	ITM2C	MAPK6	SCX
24	ORM1	MAPK13	OR11H1	COL4A1
25	APOC3	MAPKAPK5	SLC25A32	RNF213
26	HRG	MUC5B	USP17L4	PCOLCE
27	C1R	ITGB4	NANOGP8	ARGLU1
28	MT2A	FXYD3	QSER1	CORO1A
29	SAA1	FAM83H	ZNF280D	HLA-DMA
30	MT1X	IER2	POLR2J2	TYROBP
31	ORM2	PTK6	GOLGA6C	ARHGEF2
32	G0S2	AIFM3	USP17L23	NOTCH3
33	C4B	WNK4	IFNA4	MMP14
34	APOB	MYO7B	PKP1	CREBZF
35	AMBP	CLDN4	ARID3B	PDLIM3
36	APOC2	TCF25	TTC26	SMCHD1
37	HPD	COX5A	SPATA31A5	LPCAT1
38	C1S	PRSS8	DRP2	LTB
39	MAT1A	HSF1	SHLD3	PTGDS
40	ADH1B	EPCAM	INPP4B	PLEKHG2
41	LBP	BAIAP2L2	CCER2	F2R
42	ITIH3	SLC25A39	OR11H2	IGHV3-33
43	SERPINA3	FOXP4	TMED3	TNS4
44	CFH	CRACR2B	FHOD3	C1QC
45	MT1B	SREBF1	ZNF85	NES
46	APOC4	TFF3	ZNF786	CENPT
47	SLC39A14	PTPRF	DHFR2	ACTA2
48	ALDOB	TLE2	IQCH	COL6A3
49	KNG1	S100A14	DDX11	ITPR3
50	SOD2	HES6	MAP3K1	SMTN

Supplementary Table 7. Related to Figure S2

Uniquely upregulated genes of PT36 for each biologically annotated cluster (spaRNASeq)

Rank	Cancer area	Hepatocyte area	CAFs in cancer area
1	ALDH1B1	SAA2	LCN2
2	EPHB3	SAA1	PMEPA1
3	SELENBP1	FGG	QSOX1
4	POLR1D	HP	HLA-G
5	TRABD2A	CRP	RNASE1
6	SFN	ORM1	MDK
7	MAOA	FGA	GPRC5A
8	LGALS4	FGB	SPP1
9	HMGB2	C3	SEC61G
10	MAPK3	ALB	NKD1
11	PHGR1	VTN	LAMP1
12	PPP1R1B	CYP2E1	SOD3
13	CDX1	MT2A	AEBP1
14	MAPK13	HPX	NPC2
15	HSD11B2	APOA1	CEL
16	RB1	SERPINA1	MFGE8
17	FXYD3	APOC3	INAVA
18	TRPM4	RBP4	CAPG
19	NDUFB2	APOE	MTRNR2L12
20	HNRNPL	APOA2	KDELR2
21	PDHA1	CLU	SPARC
22	HNRNPD	TF	FLNA
23	SLC26A3	APOB	IGFBP5
24	SLC25A5	APOC1	TMED10
25	S100P	AHSG	BEX3
26	ATP5F1C	AMBP	TGFBI
27	PRDX5	C4B	CHPF
28	MARCKSL1	C4A	EGLN3
29	VIL1	ORM2	TXNIP
30	TMEM54	C1R	POSTN
31	LGALS3	A1BG	CREB3
32	UBE2C	PLA2G2A	COL6A3
33	ZFP36L2	ADH1B	ARHGEF1
34	HSD17B10	MT1E	TIMP1
35	MCM7	GC	IFI6
36	ATP5MC3	MT1G	ACTA2
37	MUC12	AGT	EMILIN1
38	ARPC1A	SERPINA3	COL3A1
39	COX5A	CFB	TXNDC5
40	SYAP1	ITIH4	VIM
41	NBL1	LBP	DBNL
42	PTGFRN	ALDOB	TAGLN
43	SLC12A2	ITIH3	ELOVL5
44	MPP1	ASGR1	MELTF
45	COX4I1	CP	TIMP3
46	ITM2C	ATF5	CALU
47	PRELID3B	C1S	TBL2
48	CDX2	HRG	CD46
49	PFKL	ITIH2	KLK10
50	CACYBP	HAMP	DHRS7

Supplementary Table 8. Related to Figure S2

Uniquely upregulated genes of PT44 for each biologically annotated cluster (spaRNASeq)

Rank	Hepatocyte area	Cancer area	Area of hepatic artery with lymphatic infiltration	Hepatic triads
1	GSTA1	CLDN4	IGKC	AQP1
2	ADH4	S100A6	PTGDS	KRT7
3	TTR	CLDN7	IGHG1	SFRP5
4	BAAT	GSTP1	IGHV3-30	CXCL6
5	HMGCS2	EPCAM	IGHA1	MUC6
6	CPS1	HMGA1	IGKV1D-12	DCDC2
7	HGD	CLDN3	IGLC1	ANXA4
8	BHMT	CDC42EP5	IGHV3-35	FXYD2
9	APOC3	SLC25A6	IGHM	NPW
10	ALDOB	TSPAN8	IGLC2	CHST4
11	TF	LYZ	IGKV3OR2-268	FGFR3
12	TAT	MYH14	JCHAIN	DEFB1
13	ADH1B	GPRC5A	IGFBP7	CITED4
14	CYP2A6	CDX1	COL3A1	CRIP2
15	GADD45G	S100A10	C7	ERICH5
16	ADH1A	CXCL5	CCN2	CYP3A7
17	MAT1A	SPINT2	COL1A2	HOMER2
18	AQP9	CEACAM5	IGLC3	PKHD1
19	ALDH1L1	RAB25	CCN1	CLDN1
20	DCXR	IER3	DEPP1	FOSB
21	CYP2B6	DDR1	AEBP1	AGRN
22	TTC36	NQO1	COL1A1	SLC4A4
23	CYP1A2	S100A14	ACTA2	FGFR2
24	APOA2	C19orf33	CD74	AHNAK
25	ACSL1	ITPR3	SPARC	ZBTB16
26	SULT2A1	CYP2S1	TAGLN	SORBS2
27	APOC4	ELF3	HLA-DRB1	TACC1
28	APOH	CCND2	HLA-DRA	SPP1
29	APOC1	RNF43	IGKV4-1	CFTR
30	CYP2C8	PLCB3	DPT	HNF1B
31	CYP2C9	MGAT4B	SOD3	SCD5
32	CYP2A7	CFL1	IGHV3-11	ANPEP
33	CMBL	FAM83H	DCN	NEDD9
34	ALB	PGGHG	MFAP4	LAMA2
35	AOX1	ARPC1B	MYL9	ARRDC2
36	AZGP1	EIF6	CXCR4	TACSTD2
37	SELENOP	HSPA1B	EMILIN1	UGT2B15
38	DPYS	TFF1	FLNA	SH3YL1
39	CES1	PIGR	SERPINE1	DTNA
40	AHSG	PPDPF	BGN	CCDC9B
41	HPD	PKP3	CAVIN1	BICC1
42	SDS	PHGR1	IGHV3OR16-13	MYADM
43	HRG	S100P	MYH11	OBSL1
44	ANG	PLEC	FBN1	CD59
45	PCK1	CLIC1	HLA-DPB1	CAV2
46	KNG1	MCM7	IGHG4	SERPINA5
47	HSD17B6	INF2	NR4A1	FSTL3
48	AMBP	AHCY	C11orf96	HES4
49	SERPINC1	IFI27	HLA-DPA1	CCL21
50	ASS1	SNRPD1	COL6A2	RASSF4

Supplementary Table 9. related to Figure 2

Uniquely upregulated genes of PT54 for each biologically annotated cluster (spaRNASeq)

Rank	Hepatocyte area	Invasion front	Cancer area
1	ALB	COL1A2	GOLM1
2	FGG	COL3A1	GPA33
3	CYP2A7	COL4A1	CKB
4	MT1X	SPARC	SLC12A2
5	TTR	TPM2	MIF
6	FGA	IER3	KRT20
7	TF	MCM7	SPINT2
8	RBP4	FLNA	ALDOA
9	APOE	NES	TFF3
10	APOA1	TP53I11	CYP2S1
11	APOC3	TIMP1	EPCAM
12	HP	AREG	DDR1
13	AHSG	HES4	PKM
14	HRG	LZTS3	CLDN7
15	CYP2E1	TPM1	CEACAM5
16	HPX	C7orf50	PPP1R1B
17	FGB	EPHB4	FXYD3
18	C1R	PLEKHG5	CSTB
19	CYP2B6	CDC25B	TM4SF1
20	AZGP1	NRARP	CCDC183
21	HPD	CCN2	EFHD2
22	ITIH2	CAVIN1	ANXA4
23	CLU	REPIN1	BCAP31
24	MAT1A	NR4A1	CLDN3
25	APOC1	IGFBP7	TSPAN8
26	ADH4	ILF3	HSPH1
27	MT2A	FBN1	GPRC5A
28	APOB	ACTA2	FABP1
29	C4A	LTBP4	TGFBI
30	VTN	PDGFA	S100A10
31	ALDOB	SPIRE2	PMEPA1
32	APOA2	MARCKS	VEGFA
33	AMBP	TPX2	DNAJB1
34	MT1M	PKN1	ARID3A
35	APOC4	EXOSC6	S100P
36	CYP4A11	CDK6	LGALS4
37	A1BG	PLEKHG2	BLCAP
38	IGFBP1	CDK4	CD9
39	C3	ARGLU1	HSPA1B
40	MT1E	SNX5	HSP90AB1
41	HAMP	YBX3	ATP1B1
42	TFR2	SMTN	EGLN3
43	ADH1B	ACOT7	NCBP2
44	CYP2A6	HNRNPUL1	TFRC
45	ALDH1L1	CBX4	PPDPF
46	ACSL1	ASCL2	PLAUR
47	MT1B	GRK2	SLC2A1
48	ORM1	TELO2	CPNE1
49	FBP1	DMTN	C19orf33
50	CRP	PDLIM3	PRSS8

Supplementary Table 10. Related to Figure 2

Genes uniquely upregulated in rHGP and dHGP cancer and hepatocyte areas (spaRNASeq)

Rank	rHGP-Cancer area	rHGP-Hepatocyte area	dHGP-Hepatocyte area	dHGP-Cancer area
1	CEACAM5	ALB	FGG	DPEP1
2	ACTB	APOC1	HRG	EPHB3
3	SPINT2	CYP2A7	IGKC	NUPR1
4	EPCAM	ALDOB	AHSG	TNS4
5	RRBP1	SDS	TF	PPP1R16A
6	PPDPF	TAT	C4A	SCX
7	TMSB4X	CYP2A6	G0S2	CCDC183
8	GNB2	AMBP	ITIH2	MTRNR2L12
9	ATP1A1	TTR	CLU	FAM83H
10	CST3	APOA2	C1R	HLA-A
11	S100A10	FOS	DCN	WNK4
12	FLNA	HP	CYP2E1	FKBP4
13	EFHD2	MT2A	CFB	ELF3
14	SLC25A6	ERRFI1	MCF2L	TNFSF9
15	CLDN3	ORM1	ADH4	CDK6
16	CLDN4	APOA1	CD74	CANT1
17	PRSS8	MT1E	IGHV3-30	IKBKB
18	PMEPA1	MAT1A	APOC3	AMN
19	GSTP1	FOSB	APOC4	NRARP
20	NQO1	CPS1	IGHJ6	CDHR1
21	CD9	INSIG1	AZGP1	TAP1
22	SEC61G	ACSL1	ALB	INF2
23	PRDX5	DUSP1	A1BG	PGGHG
24	HLA-C	SELENOP	VTN	SCRIB
25	BCAP31	A2M	COL3A1	HLA-F
26	KRT20	PCK1	APOE	PRR15L
27	SOD3	MT1X	ATP11A	AXIN2
28	KDELR2	APOH	AFM	SREBF1
29	NBL1	RHOB	FGA	TLE2
30	CD151	RBP4	C4B	PLEC
31	CLDN7	C3	IGHV3-35	CPNE7
32	PHGR1	SERPINA1	UBD	VPS51
33	SSR4	BHMT	FGF21	MYO7B
34	NOX1	CYP2B6	CYP4A11	JPH1
35	FABP1	AGT	C1S	GTF2IRD1
36	ARPC1B	FGB	FAM153B	FCGBP
37	S100A6	HAMP	LYZ	NPIPB4
38	TUBA1C	GADD45G	ORM2	MIB2
39	GNAS	NNMT	TTC36	CRACR2A
40	MARCKSL1	EGR1	RASD1	MUC5B
41	ADGRG1	ITIH4	APOB	ILF3
42	PYGB	ZFAND5	C1QC	TRAF4
43	SLC44A4	HPX	NAXD	CBLC
44	TXN	IGFBP4	F12	FCGRT
45	ATP9A	TF	KNG1	MYC
46	PDIA4	SAA1	SERPINE1	TELO2
47	ATP5MF	APOC3	HLA-DPB1	BOP1
48	AGPAT2	GADD45B	IGHG3	JAG2
49	C19orf33	FTL	MUC6	B4GALNT3

Supplementary Table 11. Related to Figure 3

Geneset of genes regulated by Lef1

Metabolism-associated genes downregulated upon induction of a dominant-negative isoform of Lef1 in DLD-1 colon cancer cells *

Gene Symbol	Gene Title
ABAT	4-aminobutyrate aminotransferase, mitochondrial
ACAA2	acetyl-Coenzyme A acyltransferase 2
ADAMTS17	A disintegrin and metalloproteinase with thrombospondin motifs 17
ARID5B	AT-rich interactive domain-containing protein 5B
CHI3L1	Chitinase-3-like protein 1
CLOCK	Circadian locomoter output cycles protein kaput
CPA6	Carboxypeptidase A6
DDC	Aromatic-L-amino-acid decarboxylase
DPYSL3	Dihydropyrimidinase-related protein 3
EIF4G3	Eukaryotic translation initiation factor 4 gamma 3
ELAVL2	ELAV-like protein 2
ELF1	ETS-related transcription factor Elf-1
HECW2	E3 ubiquitin-protein ligase HECW2
HOXD3	Homeobox protein Hox-D3
KIT	Mast/stem cell growth factor receptor
JAG1	Protein jagged-1
LCK	Proto-oncogene tyrosine-protein kinase LCK
LIPG	Endothelial lipase
MCCC2	Methylcrotonoyl-CoA carboxylase beta chain, mitochondrial
NFKBIZ	NF-kappa-B inhibitor zeta
NRP1	Neuropilin-1
PAX6	Paired box protein Pax-6
PCBD1	Pterin-4-alpha-carbinolamine dehydratase
PDK1	[Pyruvate dehydrogenase [lipoamide]] kinase isozyme 1, mitochondrial
PTCH1	Protein patched homolog 1
RNASEH1	Ribonuclease H1
ROR1	Tyrosine-protein kinase transmembrane receptor ROR1
SLC35D1	UDP-glucuronic acid/UDP-N-acetylgalactosamine transporter
SLC35D3	Solute carrier family 35 member D3
SLC7A7	Y+L amino acid transporter 1
SORBS2	Sorbin and SH3 domain-containing protein 2
SOX4	Transcription factor SOX-4
STK17B	Serine/threonine-protein kinase 17B
TCF4	Transcription factor 4
THAP2	THAP domain-containing protein 2
TLE4	Transducin-like enhancer protein 4
TUSC3	Tumor suppressor candidate 3
TXNDC13	Thioredoxin domain-containing protein 13
UGT2B28	UDP-glucuronosyltransferase 2B28

Note:

** references: Pate, K.T., et al. Wnt signaling directs a metabolic program of glycolysis and angiogenesis in colon cancer. EMBO J 33, 1454-1473 (2014).*

Supplementary Table 12. Related to Figure 3

Clinical Characteristics of 10 patients resected for CRCLM used for establishment of PDOs

HGP	Gender	Age at resection	Year of resection	Initial postop. UICC	Location primary tumor	MS status	KRAS mutation	RAS inhibition	Pre-treatment
rHGP	Male	72	2020	IV	Colon desc.	MSS	G12V	No	FOLFOX
rHGP	Male	54	2020	III	Rectum	MSS	No	Candesartan	FOLFOX
rHGP	Male	69	2021	III	Sigma	MSS	No	No	Oxaliplatin, Xeloda, FOLFIRI
rHGP	Female	62	2021	II	Sigma	MSS	No	Candesartan	No
rHGP	Male	77	2021	III	Sigma	MSS	Q61R	No	No
dHGP	Male	44	2020	IV	Colon asc.	MSS	No	Ramipril	FOLFOX, Cetuximab
dHGP	Female	67	2020	IV	Rectum	MSS	G12D	Enalapril	Oxaliplatin, Bevacizumab
dHGP	Female	67	2020	IV	Rectum	MSS	Gly12Val	Candesartan	No
dHGP	Male	63	2021	III	Sigma	MSS	No	No	No
dHGP	Male	66	2021	IV	Sigma	MSS	No	No	Capecitabin/Oxaliplatin; Irinotecan/ 5-FU; Bevacizumab

Note: Initial postoperative UICC describes UICC at timepoint of resection of primary tumor; Neoadjuvant treatment: within 6 months prior to CRCLM resection

Supplementary Table 13. Related to Figure S3

Clinical Characteristics of 6 patients resected for CRCLM used for scRNASeq

PT-ID	HGP	Gender	Age at resection	Year of resection	Initial postop. UICC	Location primary tumor	MS status	KRAS mutation	RAS inhibition	Pre-treatment
PT59	rHGP	Male	63	2020	IV	Rectum	MSS	N/A	Ramipril	No
PT44	rHGP	Female	69	2020	III	Colon asc.	MSS	No	No	No
PT54	rHGP	Female	53	2020	IV	Rectum	MSS	No	No	FOLFOX
PT55	dHGP	Female	67	2020	IV	Rectum	MSS	Gly12Val	Candesartan	No
PT52	dHGP	Male	71	2020	IV	Rectum	MSS	N/A	No	No
PT61	dHGP	Male	77	2020	IV	Sigma	MSS	No	No	No

Note: Initial postoperative UICC describes UICC at timepoint of resection of primary tumor; Neoadjuvant treatment: within 6 months prior to CRCLM resection

Supplementary Table 14. Related to Figure S3

Quality metrics scRNAseq, before manual quality control

patient ID	sample type	number of reads	number of UMIs	number of events	mean UMI/event	median UMIs/ event	mean genes/ event	median genes/ event	saturation
PT-44	tumor	354891250	20825636	10212	2039	181	411	104	84.0%
PT-44	adjunct normal	293845852	22644503	10219	2216	862	581	151	66.5%
PT-52	tumor	334917162	2151754	11434	188	16	50	14	98.1%
PT-52	adjunct normal	353380327	18280261	10209	1791	146	501	77	79.3%
PT-54	tumor	334728887	28872502	10167	2840	434	584	238	78.0%
PT-54	adjunct normal	350565232	16112478	10132	1590	568	559	228	78.2%
PT-55	tumor	241408127	674431	4485	150	15	63	12	98.8%
PT-55	adjunct normal	346822637	16116260	10088	1598	476	491	110	77.7%
PT-59	tumor	319404388	1215084	12440	98	18	22	13	98.9%
PT-59	adjunct normal	397074211	15422141	10363	1488	529	428	98	84.4%
PT-61	tumor	319566831	23889302	10176	2348	336	399	150	76.5%
PT-61	adjunct normal	398639591	21069016	10324	2041	196	580	104	80.6%

Quality metrics scRNAseq, after manual quality control

patient ID	sample type	number of UMIs	number of cells	mean UMI/cell	median UMI/cells	mean genes/ cell	median genes/ cell
PT-44	tumor	12110141	1026	11803	8452	2699	2587
PT-44	adjunct normal	14350664	2612	5494	3931	1709	1533
PT-52	tumor	1596960	132	12098	7563	2324	2268
PT-52	adjunct normal	14418727	1550	9302	7253	2552	2555
PT-54	tumor	15801778	7257	2177	446	581	255
PT-54	adjunct normal	11628103	2725	4267	2413	1468	1039
PT-55	tumor	15862	12	1322	716	539	359
PT-55	adjunct normal	11425470	1741	6563	5059	2052	1960
PT-59	tumor	257197	33	7794	1640	1458	458
PT-59	adjunct normal	9516955	1425	6679	5402	2152	2104
PT-61	tumor	10172530	2133	4769	1074	1113	372
PT-61	adjunct normal	17341521	1773	9781	6878	2632	2469

Supplementary Table 15. Related to Figure S3

Uniquely upregulated marker genes of main clusters (scRNAseq)

Rank	**Granulocytes**	**Cholangiocytes**	**Cancer Cells**	**Fibroblasts**	**T-Cells**	**B-Cells**	**Endothelial Cells**	**Hepatocytes**	**NK-Cells**	**Plasma Cells**
1	LYZ	ANXA4	FABP1	IGFBP7	CCL5	MS4A1	AKAP12	ALB	KLRD1	IGKC
2	HLA-DRA	SPP1	S100A6	TAGLN	IL32	BANK1	CCL14	APOA1	NKG7	IGHG1
3	CD74	TM4SF4	RPL39	CALD1	CD2	RALGPS2	LIFR	TF	GNLY	JCHAIN
4	HLA-DRB1	KRT18	RPS21	RGS5	CD69	IGHM	PTPRB	FGB	PTPRC	MZB1
5	CST3	KRT8	RPS18	ACTA2	BCL11B	CD37	DNASE1L3	APOB	CD247	IGHA1
6	CTSS	DCDC2	RPL36A	SPARC	IL7R	BCL11A	FCN3	HP	CCL4	IGLC2
7	SAT1	DST	PHGR1	C11orf96	BTG1	HLA-DQA1	CRHBP	FGA	PRF1	HERPUD1
8	IFI30	AFDN	RPL37	SPARCL1	CD3D	AFF3	FLT1	APOA2	TXK	IGHG3
9	HLA-DPA1	BICC1	TFF3	MYL9	CXCR4	SMCHD1	CAVIN2	APOC3	KLRK1	TXNDC5
10	HLA-DPB1	SERPINA3	RPS19	TPM2	TRAC	CD83	IL6ST	APOC1	ATM	HSP90B1
11	FGL2	PKHD1	RPL36	BGN	S100A4	BIRC3	PPFIBP1	APOH	KLRF1	IGLC3
12	SRGN	KRT7	RPS6	VIM	HLA-A	CD79A	TIMP3	APOE	PYHIN1	SSR4
13	AIF1	SORBS2	RPL12	COL3A1	CD3G	LTB	EPAS1	FGG	IL2RB	TENT5C
14	PSAP	ONECUT1	RPL13	IGFBP5	B2M	FCRL1	MAF	RBP4	GZMA	XBP1
15	FTH1	SOX4	FXYD3	MYH11	ZFP36L2	PRDM2	LDB2	TTR	RNF213	BTG2
16	TYROBP	DEFB1	RPS12	TPM1	HLA-B	NIBAN3	TCF4	AMBP	MYBL1	FCRL5
17	COTL1	DSP	EPCAM	COL1A2	RPS29	LY9	ADGRF5	SERPINA1	KLRB1	IGLC1
18	FTL	CLDN1	RPL37A	COL1A1	CD3E	CIITA	CD36	ORM1	FYN	TP53INP1
19	HLA-DQB1	ANXA2	RPS4X	TIMP1	FYB1	RIPOR2	INSR	CP	CD7	ANKRD28
20	VCAN	SNHG14	RPL21	MGP	TRBC2	STX7	KDR	ALDOB	TXNIP	PDK1
21	NAMPT	SLC4A4	GAPDH	MT2A	HLA-C	SP100	CTSL	VTN	CEP78	RAB30
22	S100A9	ABCC3	RPL13A	ADAMTS1	HCST	MEF2C	F8	PCK1	AKNA	POU2AF1
23	ACTB	CD24	RPL35	PDGFRB	RPS27	CD55	SPTBN1	APOC2	GZMB	IGHGP
24	RGS2	VEGFA	CEACAM5	DSTN	GZMK	ADAM28	PLPP3	C3	PLAC8	FKBP11
25	MNDA	FXYD2	RPL29	LGALS1	RPS3	POU2F2	HSPG2	CLU	ARL4C	CREB3L2
26	FCER1G	SPATS2L	RPL30	MAP1B	TMSB4X	REL	TFPI	CPS1	SAMD3	DERL3
27	NEAT1	VMP1	RPL26	ID4	HLA-E	TTN	TACC1	AHSG	IKZF3	ITM2C
28	LST1	AGT	RPLP1	CRISPLD2	CD8A	PAX5	STAB1	CYP3A5	MCTP2	SEL1L
29	PTPRE	FGFR2	RPL10	ADIRF	RPS27A	TNFRSF13C	NRP1	PLG	JAK1	FBXW7
30	SOD2	SOX9	LGALS4	COL4A2	SRSF7	PRKCB	BTNL9	TAT	UTRN	IGHG2
31	CLEC7A	ATP1B1	RPL18A	ITGB1	CLEC2D	CD22	IFI27	CFH	CCL3	CPEB4
32	SAMHD1	ATP1A1	TXN	COL4A1	RPS26	LINC02397	SEC14L1	KNG1	SPON2	SEC11C
33	FCN1	HOMER2	RPL32	CARMN	RPL41	FCMR	ARHGAP29	GC	MBP	SEL1L3
34	CYBB	FARP1	RPL7A	NOTCH3	STK17B	IRF8	MRC1	ADH1B	CST7	FNDC3B
35	TYMP	ZBTB20	RPS2	COL6A2	PIK3R1	LINC00926	RAPGEF5	FN1	ETS1	IGHG4
36	CCDC88A	PMEPA1	RPL28	PALLD	PARP8	RCSD1	NR2F1	MT-ND2	STK4	SPCS3
37	LAPTM5	ELF3	LGALS3	MYLK	RPL17	SWAP70	SLC9A3R2	MT-ND4L	CELF2	JUN
38	CPVL	SERPING1	RPLP0	SOD3	DUSP2	ITPR1	GNG11	CYP3A4	SYTL3	SLAMF7
39	PLAUR	ONECUT2	RPS15	COL6A1	CDC42SE2	P2RX5	APP	CYP2C9	SLFN5	FNDC3A
40	S100A8	AKR1C1	RPS24	IFITM3	RPL23A	MIR29B2CHG	HIPK2	A1BG	RUNX3	SELENOS
41	S100A11	TNFRSF12A	RPS16	NR2F2	CD48	CD79B	NOTCH4	ORM2	PLEK	SPCS2
42	FOS	XIST	EEF1A1	MFGE8	IFNG	FCRL2	LIMCH1	CYP2E1	MBNL1	UBC
43	LCP1	ITIH5	RPS28	DCN	CD52	IGHD	TCIM	AZGP1	FGFBP2	ELL2
44	ZEB2	KCNQ1OT1	RPL7	SYNPO2	PPP2R5C	ARHGAP24	EFNB2	HPX	IRF1	PIM2
45	CTSB	PAWR	RPS11	ID3	CALM1	MYCBP2	HECW2	HRG	RORA	HSPA5
46	MS4A7	SLC12A2	RPL35A	JAG1	TNFAIP3	ARID1B	MEG3	SELENOP	PRKCH	HDLBP
47	C1orf162	LINC02532	TPT1	LHFPL6	ARHGDIB	PARP14	WWTR1	C1S	AAK1	SEC61B
48	MPEG1	CPM	CKB	TBX2	RESF1	SMAP2	MACF1	SERPINC1	FCGR3A	OGT
49	HLA-DRB5	CLDN10	KRT19	MCAM	ANXA1	NFKBID	ADGRL4	ITIH4	CEMIP2	LMAN1
50	TMSB10	PERP	S100A10	PHLDA1	RPL19	EZR	FCN2	MT-ND1	ID2	NCOA3

Supplementary Table 16. Related to Figure S5

Marker genes enriched in clustering of cancer cells (scRNAseq)

Rank	mixed	PT44	PT54	PT54 activated	PT61 activated	PT61
1	B2M	HSPA1A	CEACAM5	FABP1	RPL30	IRS2
2	FTL	MALAT1	FABP1	RPL39	RPL8	MCF2L
3	TMSB4X	XIST	TFF3	RPL13	RPS18	NEAT1
4	ACTB	NEAT1	FXYD3	RPL37	RPL12	ARHGEF7
5	TIMP1	ARGLU1	PHGR1	RPL26	RPS6	ATP11A
6	SPP1	KCNQ1OT1	ARID3A	RPL13A	RPS27A	SOX4
7	PLA2G2A	SAT1	RRBP1	RPL32	RPS3A	ANKRD10
8	LYZ	INTS6	GPRC5A	RPL41	RPL9	NOTUM
9	S100A6	POLR2J3	CEACAM6	RPL21	RPS7	BRD2
10	S100A11	WSB1	LGALS4	RPLP1	RPL17	TUBGCP3
11	FN1	HNRNPH1	PCK1	RPL10	RPL7	HSPA1B
12	ALB	HNRNPU	ELF3	TFF3	RPL37A	HSPD1
13	SERPINA1	SQSTM1	SLC12A2	RPS15A	RPS29	ING1
14	SH3BGRL3	FOSB	IER3	RPS27	RPS2	NFIA
15	CD63	CYP3A5	FERMT1	RPS21	RPS13	MT-ND3
16	MYL12B	NUFIP2	PMEPA1	RPS28	RPS26	MT-ND2
17	IFI27	HNRNPA2B1	XIST	RPS4X	RPS12	MT-ND1
18	VIM	RNF213	DSTN	RPS19	NME2	AKAP9
19	TAGLN	SFPQ	SLC26A2	RPS14	IFITM3	PITX2
20	IFI6	LRBA	KRT8	RPL36A	SNHG6	PTPRD
21	CD74	SON	ARGLU1	S100A6	IGKC	HSP90AA1
22	TYROBP	HSPH1	NORAD	RPL28	HLA-A	KAT6A
23	EMP3	ZKSCAN1	PLCG2	RPS12	RPL5	CLK1
24	SERPINA3	SRRM2	CSNK2A1	RPL29	IRS2	AGO2
25	IL32	FTX	KIF16B	RPS16	UQCRB	HSPB1
26	HLA-B	N4BP2L2	CLDN3	RPS15	RPL27	MT-ND4L
27	S100A8	DST	CST3	PHGR1	RPL39	VEGFA
28	COL1A2	DDX3X	NEAT1	RPS11	MIF	ARID1B
29	LGALS1	PLOD3	LGALS3	TPT1	ASPSCR1	KLF5
30	APOE	RNF43	SPTBN1	RPL35	CCL4	MYLIP
31	CXCL5	CSNK1A1	TPM4	RPL18A	EEF1G	AXIN2
32	KRT7	ZBTB7A	RPL37	RPL23A	GZMA	MT-ND4
33	TPT1	SNHG14	SPINT2	EEF1A1	RPL41	ANKRD10-IT1
34	VCAN	TRA2B	CSTB	RPL36	RPLP1	RBM39
35	SRGN	FOXP1	SOX4	RPS23	RPS25	HSPH1
36	MYL12A	FOS	CKB	RPL7A	GNLY	JUN
37	IFITM3	TRIM56	YWHAB	RPL35A	FTH1	UBR5
38	CXCL8	RRBP1	SNRPB	FXYD3	RPS10	ID4
39	CCL2	PLEC	CD9	RPS24	RPL7A	MSI2
40	A2M	KMT2C	CDH17	RPL14	HSP90AA1	CHD7
41	SPARC	TAF1D	SNRPB2	RPL6	EEF1A1	HSPE1
42	MMP7	TMF1	MUC12	RPL24	RPL10	MTDH
43	FGG	PTP4A2	GSTP1	RPS17	RPS3	MALAT1
44	C3	LMNA	PTBP3	RPS6	CCL4L2	DNAJB6
45	BST2	KLF6	LDHA	GAPDH	ALDH1A1	MT-ATP6
46	KRT23	ANKRD12	EPB41L2	RPS18	NOTUM	SPTAN1
47	LAPTM5	MUC17	EDN1	RPL19	PABPC1	DNAJA1
48	FGA	DDX5	CLDN4	RPL31	RPL34	GCC2
49	PMP22	PIGR	S100A10	RPS5	RPS24	MUC6
50	MSN	CXCL5	VEGFA	RPL18	RPL3	FOXQ1

Supplementary Table 17. Related to Figure 5

Marker genes enriched in clustering of endothelial cells (scRNAseq)

Rank	Neovascular	Artery	Altered Capillary-Like	Capillary	Activated Vein-Like	Vein	Lymphatic
1	PLVAP	PODXL	MEG3	FCN3	IL1R1	MGP	CCL21
2	SPARCL1	CXCL12	AKAP12	MS4A6A	NR2F1	IL33	PROX1
3	SPARC	JAG1	HERC1	CRHBP	PRSS23	ATP13A3	DST
4	SPRY1	A2M	JUN	CTSL	LIFR	EMP1	DOCK5
5	RGCC	SLC9A3R2	SLC2A3	DNASE1L3	PTGDS	CPE	PKHD1L1
6	COL4A1	TIMP3	INTS6	LYVE1	PLAC8	ADAMTS1	MMRN1
7	COL4A2	KCTD12	ADAMTS4	FCN2	SELE	TM4SF1	SEMA3A
8	APOLD1	SEMA3G	SOX4	CLEC4G	SELP	LDB2	EPB41L2
9	ITGA6	SRP14	MACF1	CCL14	TSHZ2	ST8SIA6	LAMA4
10	COL15A1	S100A6	SRSF3	CLEC4M	PLCB1	AQP1	MAP1B
11	HSPA1A	MECOM	SYNE1	OIT3	ADGRG6	TGM2	ABI3BP
12	VWA1	GSN	BRD2	CLEC1B	SOD2	PLPP3	PTPRE
13	FLT1	CD34	NTN4	MT-ATP6	C7	CLU	CALD1
14	MCF2L	ADAMTS6	SNHG5	MT-ND3	RSPO3	ADIRF	TFF3
15	ENG	CLDN5	NRP1	STAB1	MIR99AHG	FAM107A	LIMS1
16	BTNL9	PALMD	PCDH9	SELENOP	SLC38A2	RFK	DUXAP8
17	TCF4	CAV1	IER2	MT-ND5	INMT	FOSB	OLFML2A
18	EDNRB	IFI27	HES1	FTL	PELI2	CEBPD	REEP1
19	ARHGAP29	PECAM1	MEG8	MT-ND1	RAMP3	SLCO2A1	USP53
20	IGFBP4	PLPP1	PHACTR2	MT-ND4	TAGLN	CLEC14A	LAMC1
21	HSPA1B	CHRM3	ANGPT2	MT-ND2	CRIM1	SRGN	XIST
22	JDP2	ARL15	EGLN3	MAF	CFLAR	F8	TSPAN5
23	KDR	HEG1	ATF3	MT-ND4L	DGKG	INSR	KLF6
24	CTNNB1	UTRN	ZEB2	ACSM3	TCIM	YBX3	PPFIBP1
25	IVNS1ABP	ITPRID2	NABP1	ALB	NXPE3	FLRT2	FLT4
26	SPRY4	JAG2	DAAM1	MRO	UGCG	ZFP36	MARCKS
27	HSPG2	SERPINE2	PRICKLE2	CD4	MRC1	C11orf96	TBX1
28	PDGFD	ADGRL4	C6orf62	MARCKSL1	NAMPT	ATP1A1	FILIP1L
29	GRB10	PIK3R3	DDX3Y	STAB2	RAB3C	VWF	BMP2K
30	SEC14L1	VIM	ARHGEF7	RELN	C1QTNF1	PTPRB	PLSCR4
31	ADGRL2	PCSK5	GSK3A	CXCL16	DLC1	RAPH1	ST3GAL6
32	LAMB1	EPAS1	SLC20A1	CD36	FGFR1	MMRN2	GAS7
33	RPS15	EFNB2	JAK1	LGMN	SGK1	GAS6	LGALS1
34	ACTG1	HLA-C	ZFP36L1	SLC7A8	SLC25A37	TACC1	TBL1XR1
35	TSC22D3	TMEM47	IGLC2	MTRNR2L12	ACTN1	CD9	TC2N
36	RPS12	RAMP2	SRSF7	COTL1	MEIS2	DUSP1	STX7
37	F2RL3	SYNE2	TRIM69	HIPK2	PTGIS	RASGEF1B	IGF1
38	RPL15	KCNN3	IGF1R	NR5A2	PGM5	JUNB	SNCG
39	RPS3	NES	H19	IFITM10	NEAT1	ABCG2	STON2
40	CA2	MAP4	JMJD6	NPL	VPS13A	SOCS3	ADD3
41	RPL34	KLF2	THUMPD3-AS1	FTH1	IER3	BMPER	ABLIM1
42	GNG11	CRYBG3	PRX	CD14	LRATD2	CXCL2	IGFBP5
43	DDIT4	CAVIN1	MID1	ANKS1A	PDLIM5	MT2A	DDX17
44	RPS18	FUT8	ING1	EHD3	SESN3	LMNA	RAB11FIP1
45	JCAD	PDGFB	DENND4C	FCGR2B	FGL2	MYADM	NNMT
46	NFIB	ADGRF5	SMAD6	CTSD	FUS	HIF1A	PLCG2
47	SERPINE1	NEBL	CTBP2	RFLNB	CEMIP2	NDRG1	GNG12
48	RPS8	SLFN5	LINC00963	ABCA9	LTC4S	LINC-PINT	EFNA5
49	MGLL	VEGFC	USP13	ME2	AFF1	TSPAN7	TRMT9B
50	GASK1B	CD81	PARP14	MTRNR2L8	TIMP1	IGFBP7	CRYAB

Supplementary Table 18. Related to Figure 6.

Marker genes uniquely enriched in clustering of capillaries (scRNAseq)

Rank	Altered Capillary-Like	Capillary #1	Capillary PT54 specific	Capillary PT44 specific	Capillary #2
1	ADAMTS1	CCL14	XIST	HSPH1	CLEC4M
2	EMP1	SELENOP	NEAT1	ALB	SLC40A1
3	VIM	FTL	GOLGB1	HSP90AA1	KDR
4	CLEC14A	TMSB4X	JMJD1C	DNAJB1	STAB2
5	TCF4	RPL39	PLCG2	HSPA1A	PLPP3
6	FOSB	TPT1	ZBTB16	MT-ND4L	NRP1
7	LMNA	RPS12	BTNL9	HSPD1	LGMN
8	SNHG5	FCN3	TCIM	MT-ND3	LPAR6
9	A2M	RPLP1	TACC1	EGR1	EGFL7
10	ATP13A3	RPL10	HYAL2	MTRNR2L8	OIT3
11	MGP	RPS28	KLF9	MT-ND5	MEG3
12	JUNB	RPS18	AKAP12	HSPA1B	FCN2
13	TM4SF1	EEF1A1	XAF1	MT-ATP6	MEIS2
14	INTS6	RPS23	KLF6	CHORDC1	PARP14
15	JUN	RPL28	CEMIP2	MT-ND4	FZD4
16	FLT1	RPS4X	LINC-PINT	ACSM3	CRHBP
17	DUSP1	B2M	RRBP1	MT-ND1	ENG
18	ID1	RPL34	ODF2L	MT-ND2	CD4
19	SLC2A3	RPL37	NAMPT	APOC1	NTN4
20	ADAMTS4	RPS6	SLC38A2	MTRNR2L12	ARHGAP29
21	NR4A1	FTH1	CCNL1	HSPB1	STAB1
22	SOX4	TMSB10	RPGR	ABCA9	EHD3
23	AHNAK	RPS8	SERPINB6	APOA2	MEG8
24	BRD2	RPS15A	ANKS1A	MT-ATP8	IL6ST
25	EGR3	RPL37A	NASP	ZDHHC21	ST6GAL1
26	NDRG1	SERF2	APOLD1	APOA1	ITGA1
27	ATP1A1	RPL12	KAT6A	DNAJA4	SEMA6A
28	MYADM	IFITM3	ZNF160	APOC3	RELN
29	JUND	RPS3	HECW2	AFF1	N4BP2L2
30	EPAS1	IFI27	RASGEF1B	APOE	CLEC1B
31	GAS5	RPS27	ZFP36	DNAJB4	ZFP36L1
32	NR4A2	RPLP2	LINC00472	HSP90AB1	MTRNR2L1
33	CD74	RPL30	INSR	C1QB	TGFBR3
34	ADAMTS9	RPS3A	AKAP13	TTR	NKTR
35	IRF2BP2	RPL35A	WSB1	CCDC144A	ARGLU1
36	ADIRF	RPL13	YBX3	BAG3	PRCP
37	MYH9	RPS27A	PTPRB	LAMB1	DAB2
38	BTG1	RPL3	RBM39	HSPA6	DNASE1L3
39	EFNB2	RPL41	CTTNBP2NL	ARHGEF28	TUT4
40	NFIB	RPL32	TLR4	FGB	NR2F1
41	ABCG2	RPS13	SYNE1	HSPE1	TGFBR2
42	SRSF3	RPS2	PPFIBP1	ATRNL1	MRC1
43	LTBP4	RPL26	NOSTRIN	APOH	NRP2
44	PECAM1	TIMP1	LRATD1	TF	HERC1
45	UTRN	RPS14	RBM5	DNAJB6	DDX17
46	TUBA1A	RPL7A	FKBP5	MRO	HLA-A
47	ATF3	RPL11	ZEB2	APOB	PHLDB2
48	CAV1	CALM1	PIK3C2A	ALDOB	HLA-E
49	FOXP1	RPL9	SHANK3	SESN3	MS4A6A
50	SOX18	RPL19	SMCHD1	GALNT1	ETS1

Supplementary Table 19. Related to Figure 6

GSEA capillaries (scRNAseq)

Set	Enrichment score	NES	Direction	Pvalue	Adjusted pvalue
GOBP_NEGATIVE_REGULATION_OF_GLIAL_CELL_DIFFERENTIATION	0,94674947	1,52893503	Up	0,00010787	0,00377956
GOBP_ENDOTHELIUM_DEVELOPMENT	0,82983174	1,52461864	Up	0,00010011	0,00373528
GOBP_SYMPATHETIC_NERVOUS_SYSTEM_DEVELOPMENT	0,93403199	1,51725625	Up	0,00010756	0,00377956
GOBP_LIMB_BUD_FORMATION	0,95721069	1,50998578	Up	0,00043621	0,00913182
GOBP_ESTABLISHMENT_OF_ENDOTHELIAL_BARRIER	0,85415626	1,50932408	Up	0,00010233	0,00373528
GOBP_EMBRYONIC_DIGESTIVE_TRACT_MORPHOGENESIS	0,92990523	1,50173274	Up	0,00021575	0,00578997
GOBP_ENDOTHELIAL_CELL_DEVELOPMENT	0,83697602	1,50018237	Up	0,00010148	0,00373528
GOBP_POSITIVE_REGULATION_OF_BLOOD_PRESSURE	0,88454987	1,49979699	Up	0,00010513	0,00373528
GOBP_NEGATIVE_REGULATION_OF_CELL_FATE_COMMITMENT	0,95416966	1,49338655	Up	0,00076645	0,01315289
GOBP_REGULATION_OF_ENDOTHELIAL_CELL_DIFFERENTIATION	0,86343336	1,4923024	Up	0,00010397	0,00373528
GOBP_POSITIVE_REGULATION_OF_CYTOSOLIC_CALCIUM_ION_CONCENTRATION_INVOLVED_IN_PHOSPHOLIPASE_C_ACTIVATING_G_PROTEIN_COUPLED_SIGNALING_PATHWAY	0,87233401	1,49154292	Up	0,00010481	0,00373528
GOBP_PERICARDIUM_DEVELOPMENT	0,9068561	1,48842487	Up	0,0003211	0,00739732
REACTOME_DISEASES_ASSOCIATED_WITH_O_GLYCOSYLATION_OF_PROTEINS	0,83069229	1,48488066	Up	0,00010166	0,00373528
GOBP_CELL_DIFFERENTIATION_INVOLVED_IN_KIDNEY_DEVELOPMENT	0,83433044	1,48212159	Up	0,00010207	0,00373528
GOBP_ESTABLISHMENT_OF_ENDOTHELIAL_INTESTINAL_BARRIER	0,93306935	1,48172176	Up	0,00097815	0,01546193
GOBP_COLLAGEN_CATABOLIC_PROCESS	-0,67363246	-1,73843326	Down	0,00319489	0,03149347
REACTOME_ACTIVATION_OF_MATRIX_METALLOPROTEINASES	-0,74097572	-1,75163831	Down	0,00212314	0,02461406
REACTOME_COMPLEX_I_BIOGENESIS	-0,62409761	-1,75646491	Down	0,00549451	0,04156106
GOBP_DEFENSE_RESPONSE_TO_FUNGUS	-0,76691612	-1,76430838	Down	0,00188679	0,02287482
REACTOME_ACTIVATION_OF_THE_MRNA_UPON_BINDING_OF_THE_CAP_BINDING_COMPLEX_AND_EIFS_AND_SUBSEQUENT_BINDING_TO_43S	-0,62910385	-1,78599603	Down	0,00578035	0,04276154
GOBP_CATECHOLAMINE_UPTAKE	-0,85862876	-1,80793844	Down	0,00524246	0,04075441
GOBP_RESPONSE_TO_UV_A	-0,89156767	-1,80889561	Down	0,00499376	0,03967689
REACTOME_DEFENSINS	-0,93228303	-1,81152162	Down	0,00115075	0,01696921
REACTOME_SCAVENGING_OF_HEME_FROM_PLASMA	-0,6612821	-1,8611172	Down	0,00549451	0,04156106
GOBP_MITOCHONDRIAL_ELECTRON_TRANSPORT_NADH_TO_UBIQUINONE	-0,71091673	-1,94292535	Down	0,00487805	0,03917483
GOBP_INNATE_IMMUNE_RESPONSE_IN_MUCOSA	-0,94520457	-1,94726386	Down	0,00129366	0,01807302
GOBP_ATP_SYNTHESIS_COUPLED_PROTON_TRANSPORT	-0,8388538	-1,95628449	Down	0,00204082	0,02394525
REACTOME_FORMATION_OF_ATP_BY_CHEMIOSMOTIC_COUPLING	-0,90765488	-1,98530951	Down	0,00151745	0,02008439
GOBP_MITOCHONDRIAL_ATP_SYNTHESIS_COUPLED_PROTON_TRANSPORT	-0,88928909	-2,03054453	Down	0,00175439	0,02207316
REACTOME_CREATION_OF_C4_AND_C2_ACTIVATORS	-0,90924353	-2,58129931	Down	0,00578035	0,04276154

Supplementary Table 20. related to Figure 6

Top 150 enriched marker genes of ACL endothelial cluster

Rank	Gene name	Rank	Gene name	Rank	Gene name
1	AKAP12	51	ARHGAP29	101	ABCG2
2	FLT1	52	EGFL7	102	IER2
3	PLPP3	53	EGR1	103	NOTCH4
4	TCF4	54	MAF	104	SASH1
5	CCL14	55	ETS1	105	PREX2
6	MEG3	56	TGFBR3	106	CDH5
7	INSR	57	ID1	107	NR4A1
8	PTPRB	58	BHLHE40	108	DAAM1
9	SLC2A3	59	TACC1	109	ZFP36
10	JUN	60	ITPRIP	110	CCDC50
11	CRHBP	61	GRB10	111	SPTBN1
12	LDB2	62	HSPG2	112	MIR99AHG
13	DNASE1L3	63	SRSF3	113	LIMCH1
14	F8	64	PHACTR2	114	PARP14
15	EPAS1	65	WWTR1	115	EGR3
16	MACF1	66	PLK2	116	CNKSR3
17	LIFR	67	IL6ST	117	DLC1
18	SYNE1	68	SLC38A2	118	LMNA
19	SEC14L1	69	TFPI2	119	ARHGEF12
20	HERC1	70	RDX	120	DUSP6
21	ADAMTS4	71	NEAT1	121	C6orf62
22	EMP1	72	UTRN	122	ATF3
23	BTNL9	73	FOS	123	FOXO1
24	ADAMTS1	74	HNRNPU	124	ARRDC3
25	FOSB	75	ST6GAL1	125	NRP2
26	NRP1	76	DDX3X	126	PPP1R10
27	KDR	77	SOX4	127	TGFBR2
28	IL33	78	STAB1	128	YBX3
29	RAPGEF5	79	SLC25A37	129	IGF1R
30	FCN3	80	BRD2	130	HIPK2
31	JUND	81	MEIS2	131	GBP4
32	CLEC14A	82	JUNB	132	IFRD1
33	EFNB2	83	PRSS23	133	CRIM1
34	A2M	84	DEPP1	134	IL1R1
35	MEF2C	85	NEDD9	135	MIDN
36	TCIM	86	FILIP1	136	EGLN3
37	CD36	87	PCDH17	137	MRC1
38	HES1	88	MYADM	138	EIF4G3
39	HECW2	89	SNHG5	139	EMCN
40	PPFIBP1	90	XAF1	140	SFPQ
41	JMJD1C	91	SOX18	141	SNRK
42	DUSP1	92	ARGLU1	142	MAGI1
43	SYNE2	93	CALCRL	143	SOCS3
44	TM4SF1	94	CLEC4M	144	CAVIN2
45	TFPI	95	RAPGEF2	145	ANGPT2
46	ADGRF5	96	ATP13A3	146	CDK17
47	INTS6	97	NFIB	147	MEG8
48	NTN4	98	ZFP36L1	148	LPAR6
49	F2R	99	DOCK4	149	MID1
50	GNG11	100	TGM2	150	NR2F1

Supplementary Table 21. Related to Figure 3

Primers used for rtPCR

Target	RefSeq accession	Forward	Reverse
Dkk1	NM_012242.4	5´ - AGTGTGTACCAAGCATAGGAGAAAA - 3´	5´- TTAGTGTCTCTGACAAGTGTGAAGC - 3´
Dkk4	NM_014420.3	5´ - TCACAAGGCAGGAAGGGACA - 3´	5´ - GTCGCAACGCTGGAAGATTT - 3´
Lrp6	NM_002336.3	5´- CACTTACTTCCCTGCAATTTTGAACC - 3´	5´ - TGGCCTGTAGCTGTATGACCTATG - 3´

Note: all primers supplied by IDT DNA Technologies
