# Supplementary material for: Molecular differences of angiogenic versus vessel co-opting colorectal cancer liver metastases at single-cell resolution
Source: Mol Cancer. 2023 Jan 24;22:17. doi: 10.1186/s12943-023-01713-1 (PMC9872436; doi:10.1186/s12943-023-01713-1)
Supplement: Supplementary file 2 — Additional file 2. [file 12943_2023_1713_MOESM2_ESM.pdf]

Supplementary Figure 1: Composition of clinical cohort

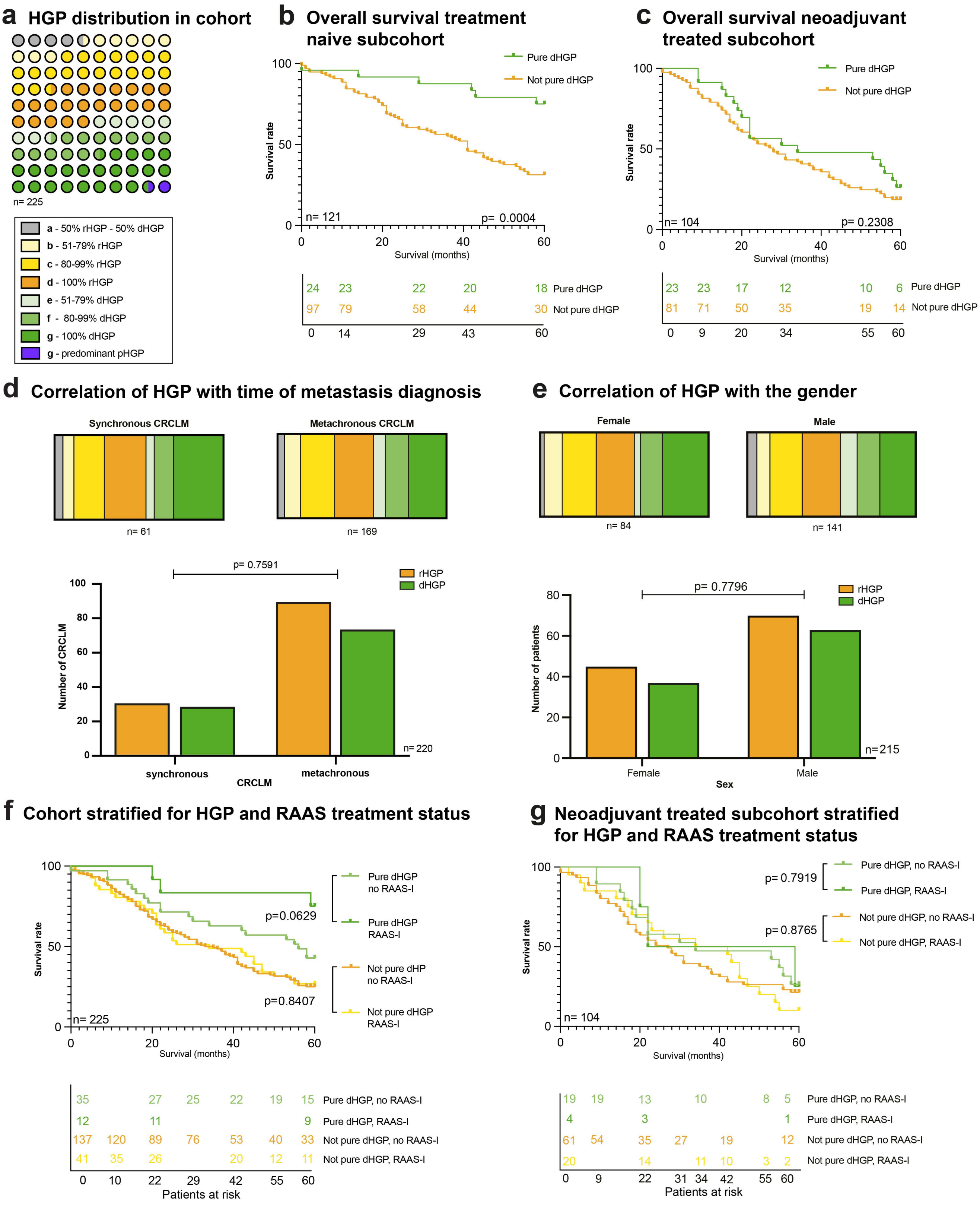

(a) Dot plot: Distribution of percentual HGP in clinical cohort (n=225). **(b)** Kaplan-Meier curves: OS after CRCLM resection depending on the HGP and treatment naive status (n=121). **(c)** Kaplan-Meier curves: OS after CRCLM resection depending on the HGP and neoadjuvant treatment status (n=104). **(d)** Bar plots: Prevalence of rHGP and dHGP in synchronous and metachronous CRCLM (n=220). **(e)** Bar plots: Prevalence of rHGP in the female and male subgroup (n=215) **(f)** Kaplan-Meier curves: OS after CRCLM resection depending on the HGP and the application of RAAS-I (n=225). **(g)** Kaplan-Meier curves: OS after CRCLM resection depending on the HGP and the application of RAAS-I in the neoadjuvant treated subgroup (n=104). P values were calculated by two-sided log-rank test.

Supplementary Figure 2: Patient-wise clustering of spots in spaRNA-seq data set

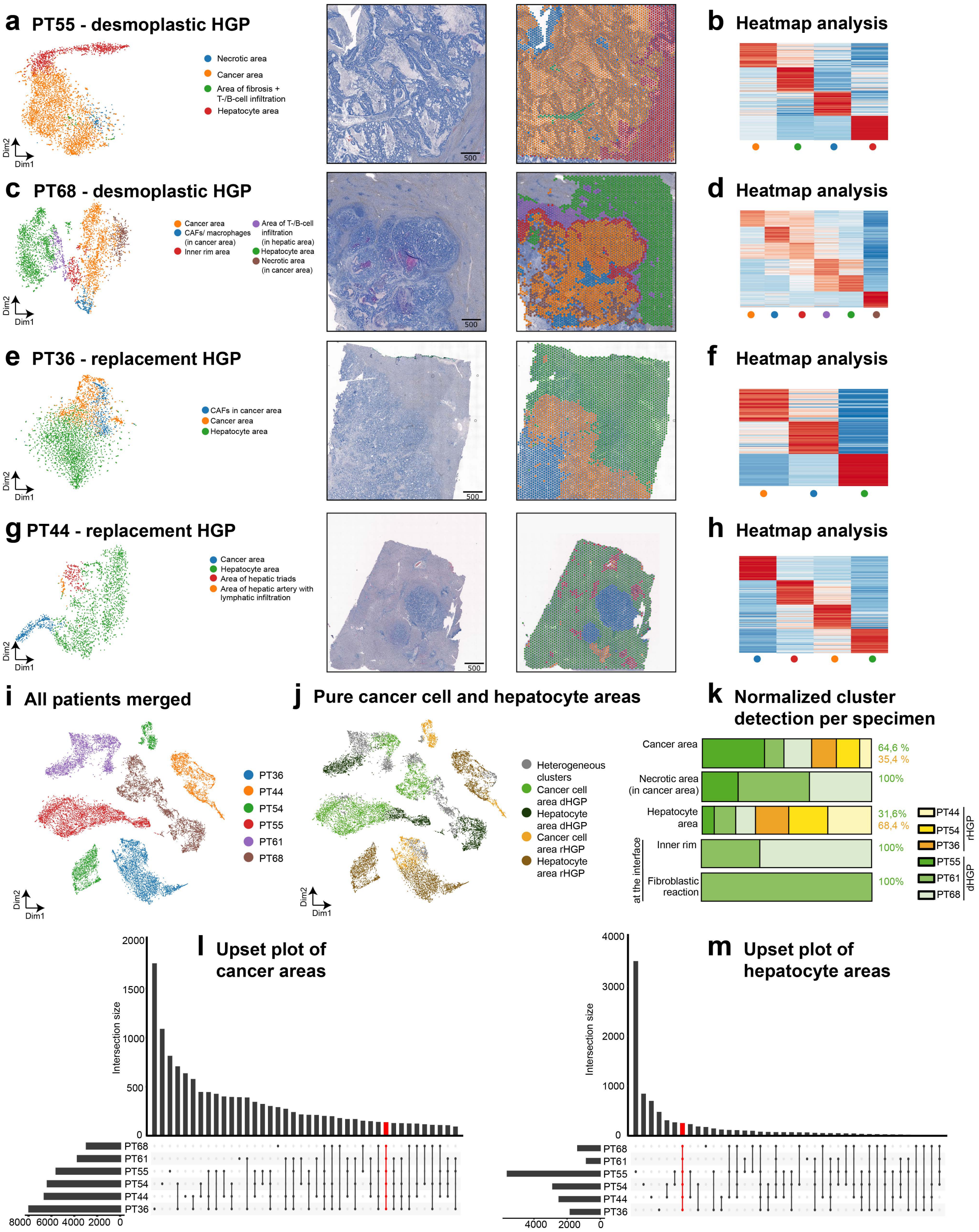

(a) t-SNE plot: 4,857 spots of PT55 (dHGP) clustered via unsupervised Louvain clustering and biologically annotated, H&E staining of PT55 and overlayed Louvain-clustering. **(b)** Heatmap of 50 uniquely upregulated genes per cluster. **(c)** t-SNE plot: 4,550 spots of PT68 (dHGP) clustered via unsupervised Louvain clustering and biologically annotated, H&E staining of PT68 and overlayed Louvain-clustering. **(d)** Heatmap of 50 uniquely upregulated genes per cluster **(e)** t-SNE plot: 3,890 spots of PT36 (rHGP) clustered via unsupervised Louvain clustering and biologically annotated, H&E staining of PT36 and overlayed Louvain-clustering. **(f)** Heatmap of 50 uniquely upregulated genes per cluster**.** **(g)** t-SNE plot: 2,843 spots of PT44 (rHGP) clustered via unsupervised Louvain clustering and biologically annotated, H&E staining of PT44 and overlayed Louvain-clustering. **(h)** Heatmap of 50 uniquely upregulated genes per cluster. **(i)** t-SNE plot: 21,804 spots of all patients annotated for patient of origin. **(j)** t-SNE plot: 21,804 spots of all patients biologically annotated. **(k)** Bar plots: normalized distribution of tissue types per specimen. **(l)** Upset plot visualization highlighting the number of genes commonly expressed in all 6 cancer areas. **(m)** Upset plot visualization highlighting the number of genes commonly expressed in all 6 hepatocyte areas.

Supplementary Figure 3: Clustering of scRNA-seq data set

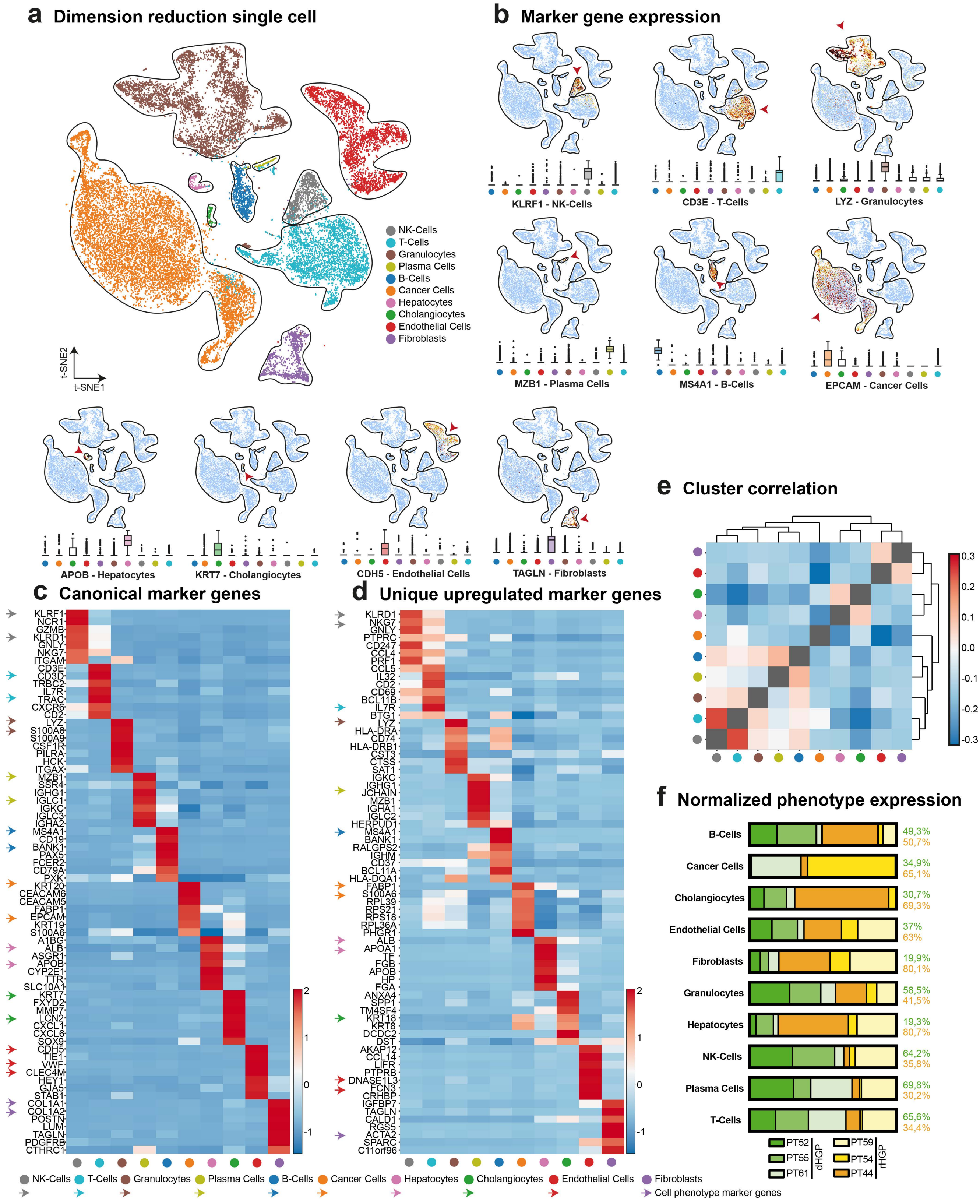

(a) t-SNE plot: 22,419 analyzed cells from tumor and corresponding healthy liver clustered via unsupervised Louvain clustering and biologically annotated. **(b)** t-SNE plots: canonical marker genes per identified cell population. **(c)** Heatmap showing expression of canonical marker genes per cluster. **(d)** Heatmap showing 10 highest uniquely upregulated genes per cluster. **(e)** Correlation heatmap of annotated clusters, hierarchical clustering location for row and column. **(f)** Bar plots: Phenotype percentages normalized to proportions of the total detected cells per patient.

Supplementary Figure 4: Subclustering of cancer cells from scRNA-seq data set

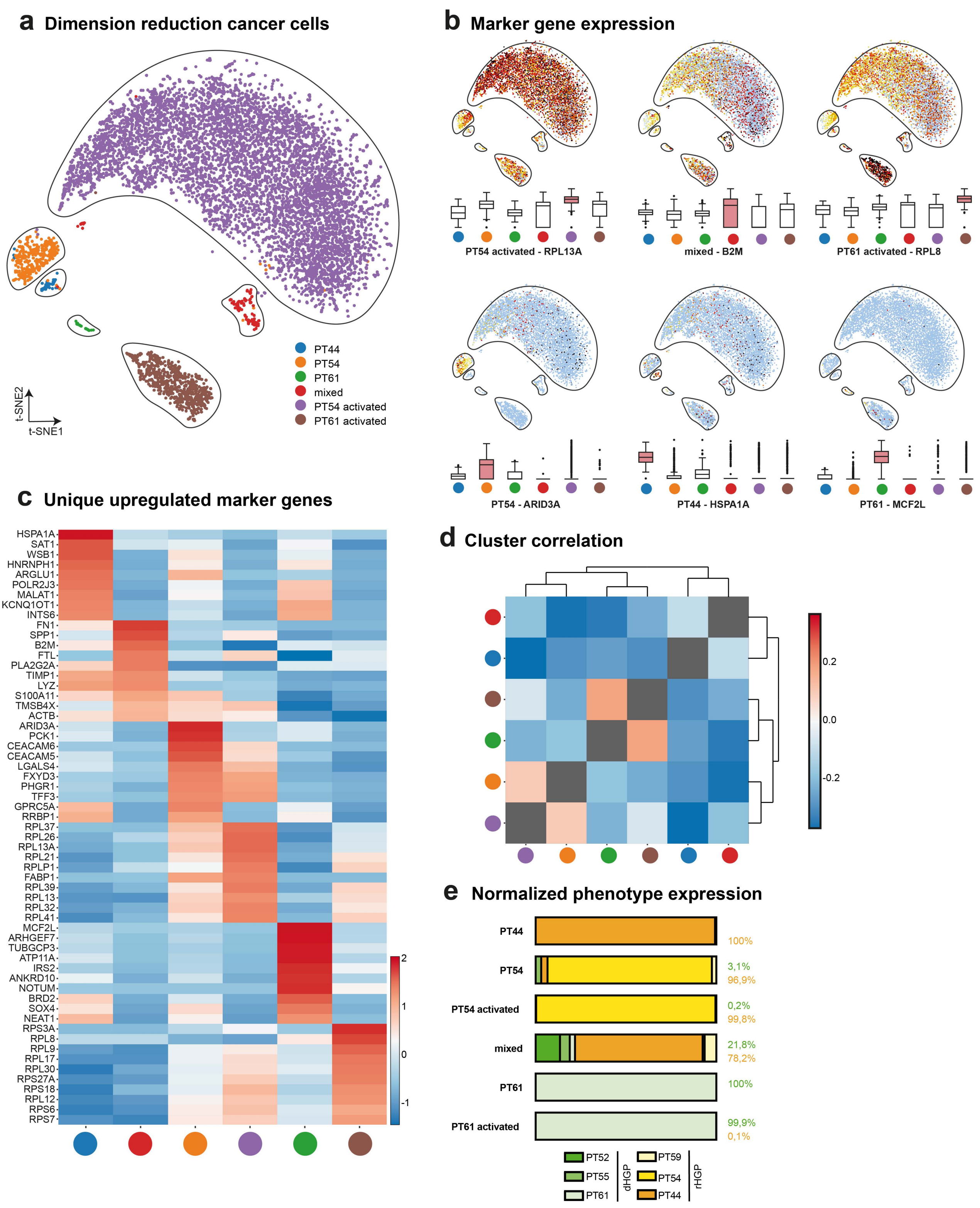

(a) t-SNE plot: 8,803 cells, previously identified as cancer cells, clustered via unsupervised Louvain clustering and biologically annotated. **(b)** Box plots and t-SNE plots: quantification for canonical marker genes of the biologically annotated clusters in Louvain clustering. **(c)** Heatmap showing 10 highest uniquely upregulated genes per cluster. **(d)** Correlation heatmap of annotated clusters, hierarchical clustering location for row and column. **(e)** Bar plots: Phenotype percentages normalized to proportions of the total detected cells per patient.

Supplementary Figure 5: Expression of metabolic genes in cancer cells from scRNA-seq data set

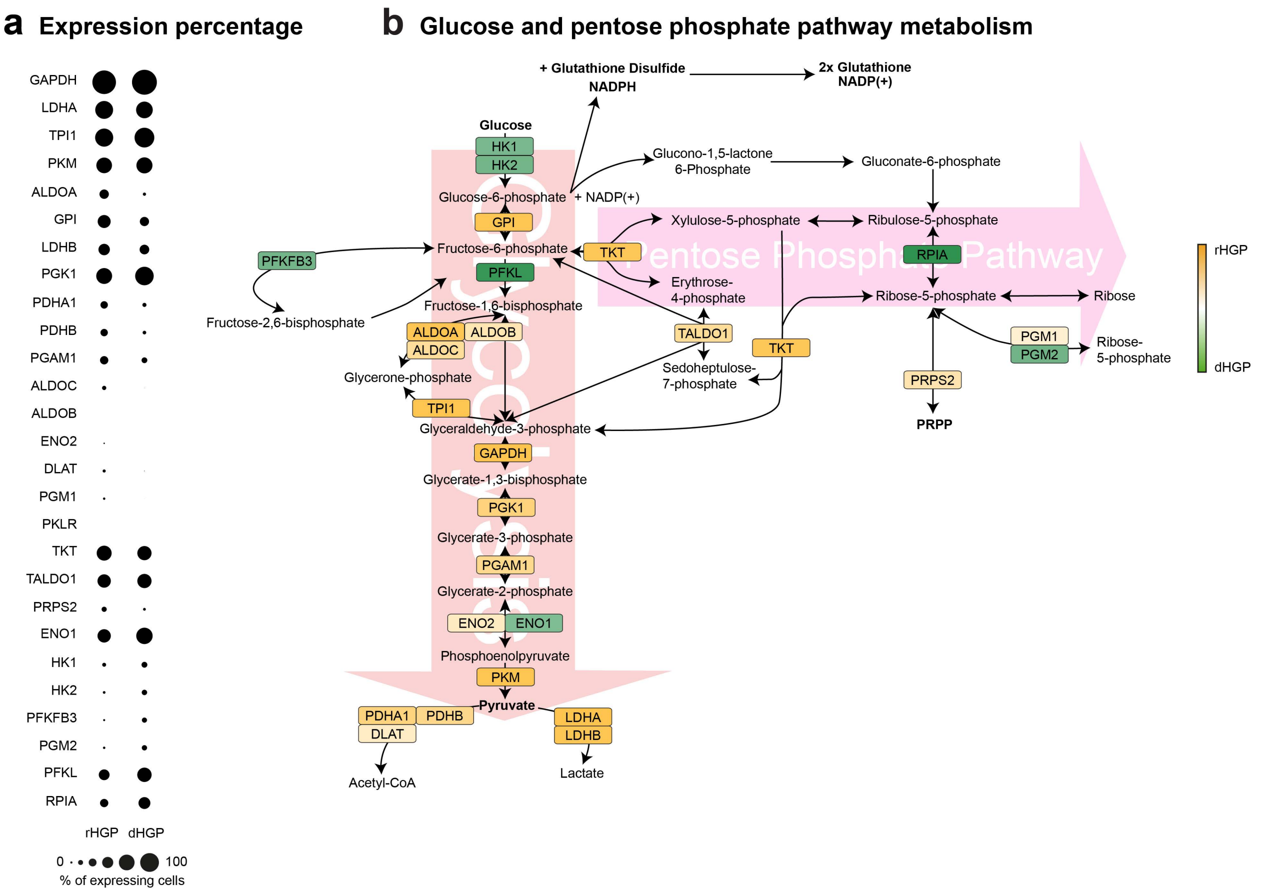

(a) Dot plot: significant differentially expressed genes between cancer cells from both HGPs in scRNA-seq data set, involved in glycolysis and PPP, mapped with expression percentages. **(b)** Pathway mapping: significant differentially expressed genes between cancer cells from both HGPs in scRNA-seq data set, mapped according to the KEGG pathway, color coded according to scaled log fold change values.
